# A Density-Dependent Switch Drives Stochastic Clustering and Polarization of Signaling Molecules

**DOI:** 10.1371/journal.pcbi.1002271

**Published:** 2011-11-10

**Authors:** Alexandra Jilkine, Sigurd B. Angenent, Lani F. Wu, Steven J. Altschuler

**Affiliations:** 1 Molecular and Cellular Biology, University of Arizona, Tucson, Arizona, United States of America; 2 Green Center for Systems Biology and Department of Pharmacology, University of Texas Southwestern Medical Center, Dallas, Texas, United States of America; 3 Mathematics Department, University of Wisconsin, Madison, Wisconsin, United States of America; University of Notre Dame, United States of America

## Abstract

Positive feedback plays a key role in the ability of signaling molecules to form highly localized clusters in the membrane or cytosol of cells. Such clustering can occur in the absence of localizing mechanisms such as pre-existing spatial cues, diffusional barriers, or molecular cross-linking. What prevents positive feedback from amplifying inevitable biological noise when an un-clustered “off” state is desired? And, what limits the spread of clusters when an “on” state is desired? Here, we show that a minimal positive feedback circuit provides the general principle for both suppressing and amplifying noise: below a critical density of signaling molecules, clustering switches off; above this threshold, highly localized clusters are recurrently generated. Clustering occurs only in the stochastic regime, suggesting that finite sizes of molecular populations cannot be ignored in signal transduction networks. The emergence of a dominant cluster for finite numbers of molecules is partly a phenomenon of random sampling, analogous to the fixation or loss of neutral mutations in finite populations. We refer to our model as the “neutral drift polarity model.” Regulating the density of signaling molecules provides a simple mechanism for a positive feedback circuit to robustly switch between clustered and un-clustered states. The intrinsic ability of positive feedback both to create and suppress clustering is a general mechanism that could operate within diverse biological networks to create dynamic spatial organization.

## Author Summary

A large body of work has focused on the ability of positive feedback in biological networks to create either switches in time (i.e., cells are either in an “on” or an “off” state) or form patterns in space (i.e., spatial organization in cells and tissues). Here, we propose a stochastic “neutral drift polarity model” by which positive feedback alone is sufficient to create switch-like behaviors both in time and space for finite molecule numbers. Our theory predicts that below a critical density of signaling molecules, positive feedback robustly maintains an off state; exceeding this threshold switches on the recurrent emergence of highly localized signaling clusters. Cluster formation requires only this minimal positive feedback circuit, and does not require additional mechanisms such as diffusion barriers, spatial cues, or biochemical inhibitors. This mechanism is general, and could be applied to a variety of cellular signaling systems to create clusters in the membrane, cytosol, or organelles.

## Introduction

The formation of local, high density regions of signaling molecules (referred to below as “clusters”) can switch cellular pathways between “off” and “on” states and direct downstream processes [1]. This transition may require careful regulation, particularly when an “on” state initiates large-scale cellular changes, such as observed in migration, cell division, or immune responses [2], [3], [4], [5], [6].

Experimental and theoretical studies have demonstrated that positive feedback plays a central role in pattern formation. Positive feedback can amplify and reinforce spatially asymmetric distributions of signaling molecules in single cells. This amplification, however, is indiscriminate; stochastic fluctuations could cause switches between “off” and “on” states to occur at undesired times, and sites of activation to occur in undesired locations [7], [8]. Additional mechanisms may be combined with positive feedback for regulating pattern formation, including coupled inhibitors [9], long-range negative feedback [10], tight regulation of input noise [11], or sequestration of components required for positive feedback [12].

Here, we wondered whether mechanisms existed within positive feedback circuits themselves to enable both the robust repression of noise required to maintain an “off” state, and the reliable establishment and persistence of distinct, high-density clusters of signaling molecules required to maintain an “on” state. First, it has been shown that positive feedback can attenuate the effects of noise. Previous studies have demonstrated that nonlinear models of positive feedback can give rise to bistable, temporal responses, which in turn set thresholds for activation below which an “off” state can be robustly maintained [13]. (The coupling of multiple positive feedback loops can also act to robustly maintain an “on” state in the presence of noisy input [14], [15].) However, these investigations were focused on temporal transitions between “off” and “on” states, and not on the emergence of spatial patterning. Second, it has been shown that positive feedback circuits can create clusters of signaling molecules through amplification of stochastic fluctuations [5], [16], [17]. In particular, discrete simulations of diffusing and interacting molecules [16], motivated by activated GTPase Ras clustering on the cell membranes of lymphoid cells [6], showed that positive feedback resulted in spatial clustering of slowly diffusing, activated molecules. In that model, clusters spread outward until the entire cell membrane was covered and the spatial patterning was lost to a homogeneous activated state. Another stochastic model [17], motivated by eukaryotic gradient sensing, showed that patches of the phosphoinositide PIP3 could accumulate near activated receptors on the surface of a cell. A coarsening process then occurred with smaller patches eventually being absorbed into larger patches. While positive feedback was shown to initiate cluster nucleation and growth in these studies, mechanisms for buffering the onset of nucleation and limiting the spread of clusters were not considered.

An important case of cluster formation is cell polarity, in which the formation of a single, asymmetric accumulation of signaling molecules, such as Rho GTPases, serves to define a unique cellular axis. Many previous theoretical studies [17], [18], [19], [20], [21], [22], [23] have provided insight into possible mechanisms by which a wide variety of eukaryotic cell types, including budding yeast, mammalian neutrophils, and amoeba can spontaneously polarize in the absence of spatial cues [2], [24], [25]. We previously considered a simple, positive feedback circuit, inspired by the ability of Cdc42 to polarize spontaneously in latrunculin-treated yeast [26]. In that model, molecules stochastically transitioned between inactive (cytosolic) or active (membrane-bound) states; and activated molecules, diffusing laterally along the membrane, recruited inactive molecules to their membrane locations. It was shown that polarity emerged from this positive feedback circuit for intermediate ranges of signaling molecule numbers. While stochastic events and diffusion eventually led to the dispersal of a cluster, at steady state the process was recurrent and a new site of polarity would eventually re-form on the membrane. In that study, the circuit operated by mass action for any fixed number of signaling molecules. However, for varying numbers of molecules, the strength of positive feedback was scaled to maintain a constant average fraction of signaling molecules on the membrane, so the circuit could not be in an “off” state. Hence, the repression of a clustered state, and transition from “off” to “on” state was not–and could not be–considered for varying numbers of molecules. Though, clustering could effectively be shut off by having so few total molecules that stochastic activation events rarely occur, or by varying other model parameters [26].

Here, in one unified model, we investigate the ability of positive feedback to reliably repress or create localized signaling domains. An essential difference between our previous and current models is that positive feedback now operates entirely through mass action kinetics (i.e. rate constants are not rescaled by total numbers of signaling molecules). In principle, removing the constraint that held the average fraction of signaling molecules constant could potentially significantly alter emergent behavior. This is indeed the case (Table 1 compares the present model to previous work, including our own, and indicates key differences in behaviors). In particular, we find that when the density of molecules is below an easily computable threshold, all signaling molecules are expected to be inactive; hence, no clusters of activated signaling molecules form, and cells are buffered against the onset of cluster formation regardless of the constant presence of noise. Above the threshold, increasing densities leads to increasing numbers of activated molecules. This process can be applied to many cell-biological settings, and we investigate clustering of molecules in the case of cell polarity, as well as for 2-D membranes or in 3-D volumes where the inactive and active forms of the signaling molecules are not segregated to spatially distinct compartments. Taken together, we find seemingly opposing effects for noise in this positive feedback circuit: at low densities of signaling molecules, biochemical noise is ignored in an “off” state; at intermediate densities, biochemical noise drives the formation of single, polarized clusters of signaling molecules to create an “on” state; and at high densities, biochemical noise overwhelms polarization to create a spatially homogeneous “on” state.

**Table 1:** Summary of models for positive feedback driven switches and resulting behavior.

Robust On/Off Switch	Clustering Behavior	Loss of Clustering	Reference
√	X	X	[6], [45]
Robust “On” Only	X	X	[14]
X	√	X	[16], [17], [22], [23]
X	√	√	[26]
√	√	√	This Work

## Results

### Formulation of positive feedback model

**Figure 1:**
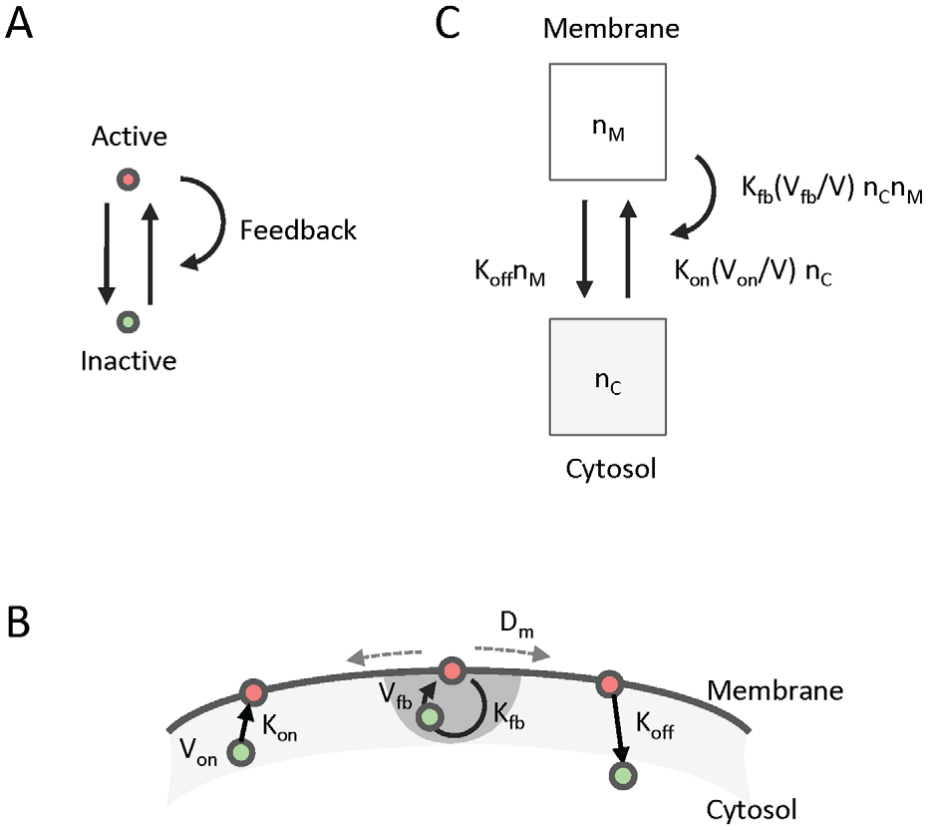
Conceptual model of positive feedback. (**A**) A simple 2-state model of positive feedback. Signaling molecules can either be in an active (red) or inactive (green) state. Molecules can transition between active and inactive states. Positive feedback occurs because active signaling molecules can recruit inactive molecules to change state. (**B**) Application of model to cell polarity. Here, active or inactive states correspond to signaling molecule localization on the membrane or cytosol, respectively. Signaling molecules may only be spontaneously activated (with rate 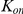), or recruited (with rate 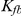) if they are within the volumes 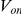 or 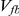 of the membrane, respectively. Active molecules can spontaneously transition to an inactive state (with rate 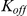). (**C**) Signaling molecule flux between the membrane and the cytosol. The total number of molecules in the membrane and cytosol are denoted by 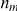 and 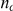, respectively. The volume of the cell is denoted by 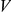.

Here, we investigate emergent behaviors of a “minimal” positive feedback circuit based on mass action kinetics interactions between two states of a signaling molecule (Figure 1A). In this conceptual model, a single molecular species spontaneously transitions between inactive and active signaling forms, while positive feedback allows activated molecules to recruit and activate nearby inactivated molecules. While many molecular networks containing positive feedback have been identified (see Table 2), detailed knowledge of their components and interactions is often incomplete. In our analysis, specific details of molecular mechanisms are elided to better focus on identifying fundamental properties of positive feedback that may be operating within diverse biological contexts.

**Table 2:** Examples of cluster formation in cell signaling systems with positive feedback.

Biological System	Geometry	Positive Feedback Loop	Parameters with Known Estimates
Cdc42 in *S. cerevisae* polarization	Membrane/cytosol	Cdc42→Cdc24→Cdc42	Diffusion rate of Cdc42 on membrane: 0.036 µm^2^/s [60]. (This estimate is 10 fold lower than in mammalian cells.) 61% of total Cdc42 is in cytosol [61].
Polarization in eukaryotic chemotaxis	Membrane/cytosol	Rac→PIP3→Rac	Diffusion of GTPase in membrane: 0.1 µm^2^/s; diffusion of GTPase in cytosol: 10 µm^2^/s [41].
EGF Receptor signaling	Membrane	Ras→SOS→Ras	Diffusion rate of Ras on membrane:0.2 µm^2^/s [62]. Ras density 4–40 molecules/µm^2^ [63].

We first consider the situation where inactive or active forms of a molecule are exclusively associated with localization to the cytosol or membrane (respectively) (Figure 1B). Here, our model is based on the following assumptions:

1. (Well-mixed cytosolic pool) Membrane molecules diffuse via Brownian motion with rate 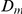, while cytosolic molecules are assumed to be distributed uniformly due to their relatively fast rates of diffusion.
2. (Mass conservation) The total number of molecules, 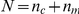, obtained by adding the number of molecules in the cytosol, 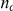, and the membrane, 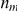, is assumed to be constant during the time frame of our observations.
3. (Mass action kinetics) Molecules can transition between their inactivated and activated forms via three mechanisms (Figure 1B). First, activated molecules on the membrane can spontaneously inactivate and return to the cytosol at rate 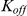. Second, inactive molecules within a cytosolic volume 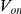 near the membrane can spontaneously activate and associate with the membrane at rate 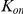. Third, inactive molecules within cytosolic “feedback” volumes 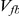 of activated membrane-bound molecules can be activated and recruited to the same membrane location as the recruiter at a rate of 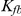. After recruitment, the recruiter and recruited molecules resume independent membrane diffusions. The fraction of molecules available for on or feedback events is scaled by 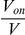 or 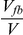 (respectively), where 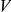 is the cell volume. Taken together, these transitions determine a model of self-interaction based entirely on mass action kinetics (Figure 1C).

We begin by discussing biological motivation for a two-state model of positive feedback. We next mathematically analyze the model described above. Finally, we discuss alternative cellular settings to the first assumption, in which the inactive and active forms intermingle in the cytosol or on the membrane, and the inactive form has a finite speed of diffusion.

### Biological motivation

This positive feedback model is applicable to diverse biological systems. In a particular biological setting, the two states of the signaling molecules could be distinguished by many mechanisms, such as biochemical modifications (e.g. phosphorylation, or GDP/GTP association) and/or cellular localization (e.g. membrane or cytosolic compartments); exchange between these forms may be regulated by additional molecular components. For example, on the membrane, the activated small GTPase Ras is observed to form dynamic nanoclusters [27], [28]. Ras activation *via* the Ras activator SOS (Son of Sevenless) has been demonstrated to contain a positive feedback loop [6]. In the nucleus, unphosphorylated splicing factors (SFs) self-organize into dynamic nuclear speckles. Speckle formation is modulated by the self-interaction (binding) of slow moving unphosphorylated SFs, whereas self-interaction is diminished in the fast-diffusing, phosphorylated state [29], [30], [31], [32]. Finally, within a cell, clustering may involve molecules cycling between membrane and cytosol. Examples include myristoylated alanine-rich C kinase substrate (MARCKS) proteins [33] that colocalize with patches of PIP2 on the plasma membrane in their dephosphorylated form [34].

An important biological application for our model is provided by proteins involved in cell polarity, such as Cdc42 and other Rho family GTPases. Like other GTPases, Cdc42 cycles between an active GTP-bound form (that is localized to the membrane) and an inactive GDP-bound state (that can be on the membrane or in the cytosol). GDI (guanine dissociation inhibitor) molecules extract the GDP-bound form of Rho family proteins from the membrane to the cytosol, where they are sequestered in an inactive pool. In the budding yeast, *Saccharomyces cerevisiae*, active Cdc42 localizes to a single zone on the plasma membrane marking the bud assembly site [2]. Dynamic Cdc42 GDP/GTP cycling is required for the polarization response in *S. cerevisiae* [35], [36], and continual exchange between membrane and cytosol is hypothesized to be essential for generating robust cell polarity [19], [20], [26].

Cycling of Cdc42 between the membrane and cytosol may be described by our two-state positive feedback circuit. First, active Cdc42 promotes the recruitment of more Cdc42 to the polarity zone, resulting in a positive feedback loop (reviewed in [2]). Second, on the timescale of cell polarization, the amount of Cdc42 can be considered to be roughly constant. Third, the inactive cytosolic pool can be considered well mixed assuming: (i) the amount of GDI in the cell is not a limiting factor [37], [38]; and (ii) switching between membrane and cytosolic states is rapid [39]. Then, we can use the fast exchange (rapid equilibrium approximation) between the membrane and cytosolic GDP-bound forms to derive a net “effective diffusion coefficient” [40] for the inactive forms. This coefficient is given by a weighted average of the membrane and cytosolic diffusion coefficients 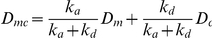, for membrane association and dissociation rates 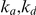 and membrane and cytosolic diffusion coefficients 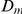 and 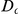. The diffusion rates in the membrane are typically 100–1000× slower than in the cytosol [41], resulting in 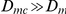. Thus, Cdc42 satisfies all three criteria of our positive feedback model. More generally, this simple two-state model could be used to approximate membrane/cytosol cycling of Rho GTPases or other signaling molecules, where an active membrane-bound form undergoes slow diffusion, while an inactive cytosolic pool is well mixed.

### Numerical exploration of model behaviors

To provide insight into emergent model behaviors, we made use of numerical simulations. We first simulated the positive feedback circuit for a 1-D circular membrane to facilitate easy visualization of signaling molecule behaviors in time and space. We quantified the total number of signaling molecules on the membrane as well as the frequency of polarization, determined by whether ≥50% of the molecules were contained within a contiguous region (ranging from 15 to 25%) of the membrane.

**Figure 2 pcbi-1002271-g002:**
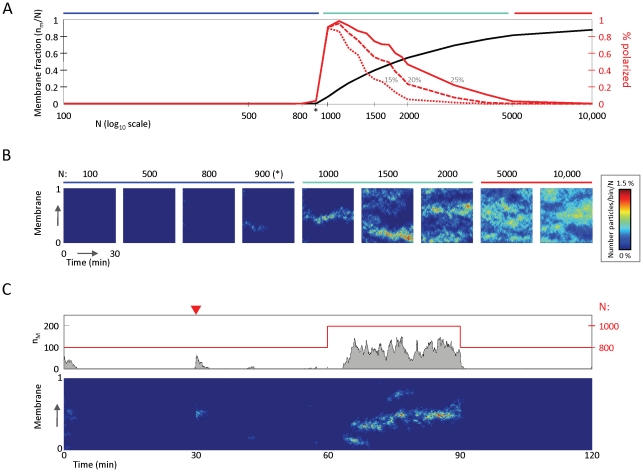
Repression, emergence, and loss of polarity for increasing concentrations of signaling molecules. (**A**) Three regions of polarization behavior are shown: repression (blue bar); spontaneous emergence (cyan bar) and loss (red bar);. Black curve: averaged membrane fractions of molecules. Red curves: averaged probabilities of observing polarization; signaling molecules are considered clustered when more than 20 molecules are present on the membrane, and 50% of all molecules on the membrane are within a small region covering 15% (dotted curve), 20% (dashed curve), or 25% (solid curve) of the membrane. (*) indicates critical number of molecules 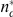. Results are averaged of 50 simulations, performed for each indicated value of 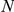. Changing the minimum cluster size to 10 molecules from 20 does not affect results (not shown). (**B**) Kymographs of simulations for values of 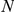 chosen from the three regions shown in (A). (**C**) Positive feedback circuits give rise to switch-like behaviors in time and space. 0 min: the positive feedback circuit is initialized with 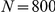 molecules, 10% of which are randomly distributed on the membrane, and polarity is repressed (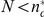); 30 min (red triangle): 10% of the cytosolic molecules are reseeded to 10% of the membrane; 60 min: 200 particles are added to the cytosol, and polarity switches on (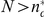); 90 min: 200 particles are removed from the cytosol and polarity switches off. Bottom panel: kymograph of simulation is as in (B); top panel: total number of molecules on membrane (gray curve and left axis) and total number of molecules in cell (red curve and right axis). Simulations were performed on a 1-D circular membrane (see Table 3 for model parameters).

To compare model behavior with our previous study of stochastic polarity [26], we varied the total number of molecules, 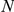 (see Table 3 for model parameters). Cells were initially seeded with 10% of the molecules randomly position on the membrane. Consistent with previous findings, for large 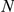 the distribution of activated signaling molecules was largely homogeneous while for intermediate 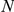 self-organized clustering occurred. However, in contrast with our previous model (Figure S1), we observed a clear “off switch”: below a critical number of signaling molecules, all molecules were localized to the cytosol; above this critical number clustering occurred (Figure 2A–B).

**Table 3:** Parameters used for simulations in Figure 2.

Parameter	Meaning	Value	Justification
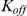	Spontaneous off rate	9 min^−1^	[26], calculated from [36].
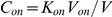	Spontaneous on rate	0.0005 min^−1^	Assumed as in [26].
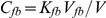	Feedback rate	0.01 min^−1^	Calculated from 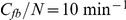 in [26].
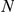	Total number of molecules	Varied	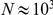 assumed in [26].
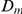	Rate of diffusion for active molecules	1.2 µm^2^ min^−1^	[26], calculated from [60].

To test the robustness of repression below this threshold, halfway through a numerical simulation we abruptly moved 50% of the molecules in the cytosolic pool to a small region covering 10% of the membrane, then restarted the simulation (Figure 2C). Again, below the critical number, polarization was always immediately lost after restimulation, indicating that the maintenance, as well as the establishment of polarization is prevented. Finally, we varied the level of “input noise” to the system by varying the spontaneous on rate 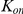 over 5 orders of magnitude. Throughout this range, a switch-like transition between no-clustering and clustering was observed (Figure S2). Thus, these simulations suggested that as 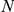 increased the behaviors of the positive feedback model transitioned from the repression, to the emergence, and finally to the homogenization of polarization.

### Mathematical model for density of molecules in the cytosol

With these observed behaviors as motivation, we next describe the mechanisms that underlie the behavior of this model in three steps. First we consider the time evolution of the number of particles in the cytosol, 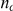. Assuming that this number can be treated as a continuous variable, whose evolution is determined by a differential equation, we find switch-like behavior. Namely, when the density 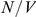 of particles in the cell lies below a certain critical value 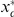 *all* particles remain in the cytosol, thus preventing polarization of the membrane; for larger values of the density 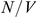 a fraction of the particles will move to the membrane, enabling polarization. Second, we remove the assumption that 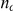 is a continuous variable, and more accurately model the number of cytosolic molecules in terms of a stochastic process. We again find that switch-like behavior emerges (Protocol S1, Section 4). Third, to determine the range of parameters in which polarization occurs, we extend the stochastic model to include the membrane-bound particle positions.

Assuming that 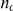 can be described by a continuous variable, our model dictates that its rate of change (Figure 1C) is given by:
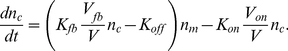
(1)We may also keep track of the density of molecules in the cytosol, 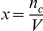. Rescaling time (so that 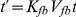), equation (1) can be rewritten more simply as:
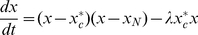
(2)where the constants are given by:
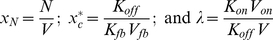
(3)The first constant, 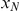, is the total density of molecules in a cell; since some molecules may be membrane-bound, 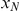 is the upper bound for the cytosolic density 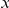 (that is, 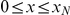). The second constant, 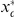, also has dimensions of density, and is dependent on parameters of the positive feedback system itself, but is independent of the cell's volume or the number of molecules it contains. The third constant, 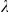, is dimensionless and reflects a ratio of spontaneous on-to-off rates. As will be shown subsequently, these three constants play critical roles in determining when polarization can and cannot occur.

### Steady state analysis

Polarity cannot emerge when the spontaneous on rate is comparable to the off rate: high molecular flux due to undirected, spontaneous on-events will overwhelm the ability of positive feedback to create localized regions of high density [26]. Thus, we analyze the system behavior when the spontaneous on rate is small relative to the off rate, that is, when 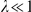. By considering the steady states of equation (2), we can understand why the “off” state is buffered from noise in the small molecule number regime.

**Figure 3 pcbi-1002271-g003:**
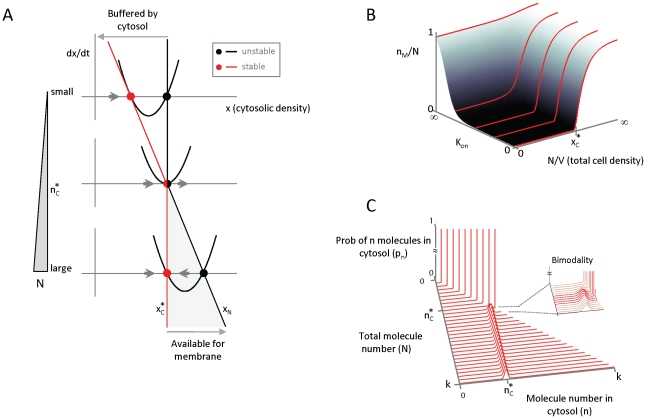
Polarity is repressed below a critical total density of signaling molecules. (**A**) Illustration shows stability of equilibrium values for cytosolic densities, 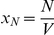 and 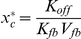, for varying 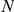 when 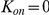. Red: stable root; black unstable root. (**B**) Shown is equilibrium membrane fraction 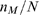 of signaling molecules on the membrane 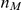 for various cell densities and different values of 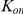. Cytosolic buffering occurs when 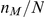 is nearly zero. (**C**) The probability, 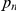, that the: cytosol contains exactly 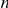 molecules is shown for different total molecule numbers, 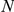, scanned between 0 and 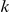. Steady state probabilities of molecule numbers in the cytosol are computed from stochastic master equation (Protocol S1). Inset: zoom-in of transition region showing bimodality of probability distribution.

When there are no spontaneous activation events (i.e., 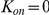, the evolution of the cytosolic density given by (2) reduces to:
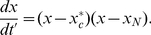
(4)The right hand side is a simple quadratic expression. Hence, at steady state, the cytosolic density is predicted to be at one of two values, 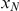 or 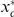, defined in equation (3). If 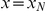, then all molecules are sequestered in the cytosol (i.e., 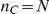). No molecules are available for the membrane, and clustering is repressed by default. If 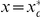, then 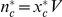 molecules are in the cytosol and the remaining 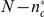 molecules are on the membrane. Then, the circuit is in a permissive state for clustering, and whether or not clustering can occur is determined by other relationships among the parameters [26]. It follows from equation (4) that the smaller of these two steady states is stable, while the larger is unstable (Figure 3A).

What determines which of these steady states a cell will be in? A key distinguishing factor is that 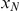 depends on the molecule number 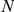 whereas 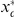 does not. This makes 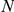 a natural parameter to vary, whose effects can easily be observed by comparing the ratio of the two steady state roots, 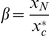 (analogous to the basic reproductive ratio [42] in theories of epidemiology, discussed later). As 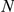 increases from small to large values, clustering goes from being repressed 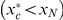 to being possible 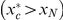 (Figure 3A). Switching occurs at the “critical” density 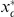 (in a so-called transcritical bifurcation) when the two roots are equal (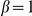). (Note that density-dependent switching is not observed if feedback is scaled to maintain a constant fraction of activated molecules [26]; the analogous ratio of roots 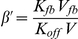 is independent of 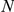.) What other parameters of this positive feedback could cells modulate to regulate repression of clustering? As can be seen from 
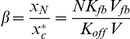
, decreasing the positive feedback rate or the recruiting volume, or increasing the membrane dissociation rate or the cell volume will expand the range of densities where clustering is repressed. Taken together, when molecular density is below an easily computable threshold, a cell will be in a repressed state for clustering.

The “off” state can also be buffered when spontaneous activation events are allowed to occur (i.e. 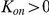) (Figure 3B). Equation (2) has two distinct steady states, given by solutions of
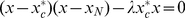
(5)When spontaneous activation events are relatively infrequent, i.e. when 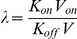 is small, we expect the roots of equation (5) to be close to the steady states of equation (4). For equation (5), the smaller root is always smaller than 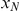 and 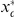, and corresponds to a stable steady state, while the larger root is always larger than 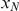 and 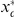, and corresponds to an unstable steady state. Because the larger root is always greater than 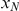, only the smaller root is physically relevant (there cannot be more particles than 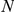). There is no exchange of stability, and equation (2) has a unique, stable steady state. However, when 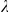 is small, the smaller root still exhibits switch-like behavior near 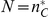 (due to the proximity of the bifurcation point; this phenomenon is described in bifurcation theory as an imperfect transcritical bifurcation with 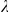 as imperfection parameter [43]).

### Stochastic model for molecule density in the cytosol

Of course, the actual number of molecules in any given cell is finite. It is known that stochastic fluctuations may drive cellular behavior to new dynamic states not seen in deterministic models [26], [44], [45], [46]. We next investigate whether the switching behavior shown in the continuous setting would also hold in a stochastic setting.

A more detailed description of the probabilities for the time evolution of the number 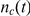 of molecules in the cytosol is given in terms of a one-step continuous time stochastic process [47]. In a stochastic process, the steady state is described by its stationary distribution. This distribution specifies the probability, 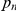, that the cytosolic pool contains exactly 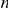 molecules for a randomly chosen cell from a large ensemble of cells, or for one cell inspected at a randomly chosen time from a sufficiently long time interval. The stationary distribution is obtained by solving the master equation (Protocol S1, Section 4 and [47]).

The switching in the preceding continuum approximation also appears in the stochastic analysis when the ratio of on-to-feedback rates, 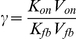, is small. We find that the stationary distributions undergo a qualitative change as 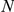 increases above 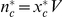. More precisely, when 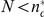 all probability density centers around 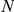, while for 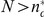 the stationary distribution 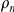 is essentially a Poisson distribution with expectation 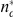 (Figure 3C). Interestingly, the stationary distribution shows bimodality near the transition point (Figure 3C, inset) while the deterministic solution is unimodal. The ability of stochasticity to induce bimodality to a deterministic mass action equation was also recently reported for cellular signaling in phosphorylation-dephosphorylation cycles [45]. As we discuss next, the ratio 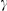 cannot be too large if clustering in the “on” state is also desired.

### Polarization of signaling molecules

The preceding analysis focused on the overall numbers of molecules in inactive or active states. We next examine the spatial distribution of the active signaling molecules. We perform this analysis in the stochastic setting, as the continuous setting modeled by partial differential equations leads to a homogeneous steady state (Protocol S1, Section 6). The basis for stochastic cluster formation, previously described [26], [48], remains valid in this current work for any specified set of parameters. However, the feedback reaction rate in the present work depends differently on 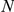 and, as a consequence, the range of parameters that permit polarization are changed. Here, we provide a new approach for analyzing the mechanism responsible for the emergence of clusters and calculate the parameter ranges in which polarization is possible (Protocol S1, Section 5).

The phenomenon of polarization is defined by the property that a large fraction of the membrane-bound signaling molecules cluster within one small region of the membrane. In general, molecules will be distributed unevenly on the membrane due to stochastic fluctuations. Recruitment and disassociation will not deterministically amplify such asymmetries; at equilibrium, each membrane-bound signaling molecule will recruit or disassociate with equal probabilities causing these effects to cancel at first approximation. However, the stochastic nature of the process can cause imbalances in the molecular distribution to undergo a neutral drift in which eventually a small region of the membrane contains most molecules while the remainder is largely depleted.

**Figure 4:**
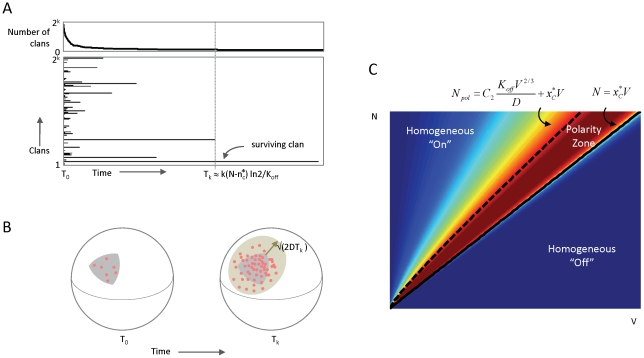
Positive feedback can recurrently generate a single, polarized cluster of signaling molecules.

When 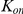 is small, on-events are infrequent and we can analyze the clustering mechanism in between on-events by grouping the membrane-bound signaling molecules into “clans” and tracking their genealogy [26]. Initially the clans are defined by dividing the membrane into a large number of small regions and declaring all molecules in any such region to form one clan. Clan genealogy is defined by assigning each newly recruited molecule to the clan of its recruiter, and by erasing the clan identity of any spontaneously dissociating molecule. As time progresses, clans will shrink and grow in population size, but once a clan has lost its last member it becomes extinct and cannot return. As long as no on-events occur, the number of clans cannot increase. Our analysis shows that if the time interval between two on-events is sufficiently long then, the expected time in which only half the original clans survive is (Protocol S1, Section 7)
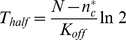
(6)If the membrane is initially partitioned into 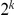 clans, then after time
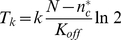
(7)only one clan is expected to remain (Figure 4A).

A range of parameters for which single clans emerge within localized domains can be computed explicitly, even when a small number of on-events are allowed (Protocol S1, Section 5). First, the frequency of on-events must be low enough that there is enough time to allow all clans but one to become extinct before the on-events significantly contribute to a new fraction of membrane-bound population of molecules. If
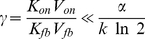
(8)then all but a (small) fraction 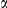 (e.g. 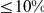) of the total molecules on the membrane will belong to a single clan. Second, if membrane diffusion is slow enough then all members of the surviving clan will be located in a small neighborhood of the site of its ancestral clan (Figure 4B). This will be true if the number of molecules is bounded by
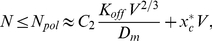
(9)where 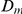 is the membrane diffusion constant and the constant 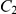 depends on the size of the initial 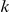 regions (Protocol S1, Section 5). Taken together, estimates (8) and (9) indicate when membrane-bound molecules will have redistributed with high probability to form a single localized cluster (Figure 4C). Note that equation (8) is a conservative estimate; polarization was observed even when 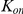 was above this bound (Figure S2).

### Numerical simulations in alternative cellular settings

**Figure 5:**
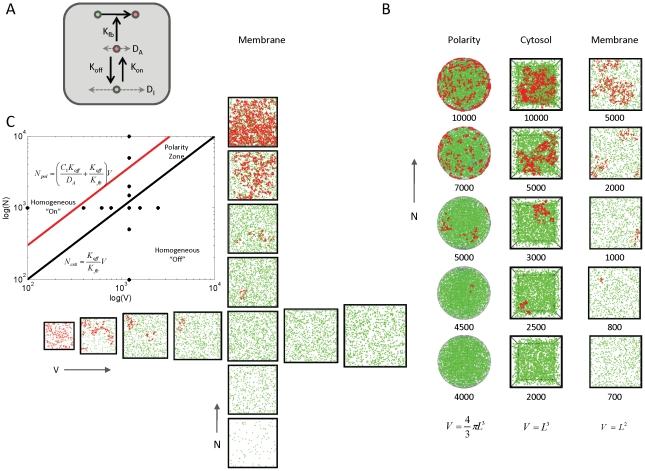
Density-dependence of spatial clustering is observed for different spatial geometries. (**A**) Modification of model shown in Figure 1B, removing the assumptions that the active and inactive molecules are spatially segregated into different spatial compartments, and that the inactive form is spatially homogeneous (infinite rate of diffusion). Here, both the active (red) and inactive (green) molecules can occupy the same compartment and diffuse at finite speeds given by rates 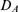 and 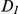, respectively. (**B**) Numerical implementation of the modified model shown in (A) for three different spatial geometries: (i) active molecules reside on the surface of sphere, while inactive molecules reside in the interior (polarity); (ii) both active and inactive molecules reside in a 3-D volume (cytosol); and (iii) both active and inactive molecules reside on a 2-D surface (membrane). For all geometries, we observe progression from buffered off state to localized clusters to homogeneous on state as the number of molecules is increased. (**C**) Phase plane diagram for the 2-D model as a function of molecule numbers and membrane area. Numerical simulations using the stochastic molecule simulator Smoldyn illustrate a density-dependent switch in clustering behavior. Inset: analytically computed phase plane diagram; “+” marks indicate locations of simulations; *V* in equations has dimensions of area (see labels at bottom (B)). All simulations in (B–C) were performed using the Smoldyn stochastic molecule simulator version 2.15 [51]; . Shown are results from running the stochastic simulation for 100 time units (see Protocol S1, Appendix for code and parameter values).

Finally, we tested applications of this positive feedback circuit to generate molecular clusters in 2-D or 3-D cellular settings, motivated by possible applications of our model framework to clustering on membranes [49] and in the nucleus [50]. We used the freely available spatial stochastic particle simulator Smoldyn version 2.15 [51] to implement an alternative version of this circuit (Figure 5A; see Protocol S1, Section 8 for differences). In particular, we removed the assumption that the inactive form exists in a spatially homogenous pool, and assumed a finite rate of diffusion, 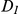, for the inactive molecules, which was still faster than the diffusion rate 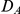 of the active molecules (i.e., 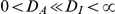). We tested the model in three different biologically motivated spatial settings, in which: (1) active molecules diffuse on the surface of a sphere, and recruit inactive forms from its interior; (2) active and inactive molecules both diffuse within the same 2D compartment, such as plasma membrane; and (3) active and inactive forms both diffusing within the same 3D volume, such as within the nucleus. In all settings, we observed transitions from a buffered “off” state, to one or several localized clusters, to a homogeneous “on” state as the number of molecules increased (Figure 5B and Video S1). For a fixed, intermediate number of molecules we observed competition between clans, until a single recurrent cluster remained (Video S2). Adjusting for the dimensions of the model parameters 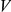 and 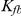 (see Protocol S1, Section 8), the transitions for this alternative model (Figure 5C and Figure S3) were in close agreement with the analytically computed phase plane.

## Discussion

It has long been appreciated that positive feedback plays a key role in intracellular signaling [13], [52] and, in particular, the ability of molecules to self-organize into highly localized clusters in the membrane or cytosol of cells. Positive feedback can cause clustering to occur in the absence of localizing mechanisms such as pre-existing spatial cues (e.g. chemoattractants), diffusional barriers (e.g. septins at the base of the primary cillium) or molecular cross-linking. What prevents positive feedback from amplifying inevitable biological noise when an un-clustered “off” state is desired? And, what limits the spread of clusters when an “on” state is desired? In theory many additional mechanisms could be postulated. Here, we find that a minimal model of a positive feedback circuit has the intrinsic ability both to suppress and amplify noise: below a critical number of signaling molecules, clustering switches off; above this threshold, highly localized clusters are recurrently generated.

Interestingly, positive feedback only produces spatially localized clusters in the stochastic regime, when one assumes a finite number of molecules and the presence of biological noise. In a continuum limit positive feedback alone is not sufficient for pattern formation, and many reaction-diffusion models have shown the need for additional mechanisms, such as long range negative feedback or substrate depletion [9], [10]. The loss of small clusters and emergence of a dominant cluster for finite numbers of molecules is partly a phenomenon of random sampling, and is somewhat analogous to the fixation or loss of neutral mutations in finite populations [53]. That clustering may not be observable in the continuous limit [26] suggests, when analyzing signal transduction networks, finite sizes of populations cannot be ignored. In homage to classic work in population genetics, we refer to our model as the “neutral drift polarity model.”

Similar threshold behaviors in autocatalytic processes appear in diverse settings [54]. Interestingly, our minimal model of positive feedback can also be recast as a well-studied mathematical model for the spread of an epidemic. In this setting: the cytosolic molecules correspond to susceptible individuals (S); membrane-bound molecules correspond to infectious individuals (I); and the recruitment of new molecules by membrane-bound molecules corresponds to the spread of infection when an infected and a susceptible individual come in contact. Collectively, these interactions are referred to as an SIS model, and form a system of equations similar to ours [55]. The dimensionless parameter 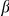, described earlier, can be interpreted as the expected number of susceptible individuals that can become infected though contact with an infected individual [42]. Our results are analogous to the property that when 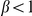 the spread of infection is repressed, whereas for 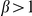 the disease is endemic in the population. The differences between the deterministic and the stochastic SIS model in a spatially homogeneous setting have been well-characterized [56], [57], [58]. In particular, the endemic steady state is only quasi-stationary, as 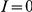 is an absorbing steady state (once there are no infective individuals left, there is no new source of infection), and stochastic fluctuations always result in eventual disease extinction [59]. Our positive feedback model differs from the SIS model in the inclusion of spontaneous transitions from the susceptible (inactive) pool to the infectious (active) pool. This significantly changes the long-term behavior of the system. Our results suggest that by including the effect of variable spread rates for susceptible and infectious individuals, introduction of new infections, and finite population sizes, parameter regimes exist in the SIS model where recurrent spatial patches of infected individuals can occur.

The neutral drift polarity model considered in this paper is a simple conceptual model that encapsulates the mechanism for particle clustering. Although this model captures the generic features of the emergence of cell polarity, and can be mathematically analyzed both in the deterministic and the stochastic regime, simplifying assumptions were made for mathematical tractability. First, in our theoretical treatment we assumed that the inactive forms are well-mixed throughout the cell. This assumption was weakened for the Smoldyn implementation, where a high but finite rate of diffusion was assumed for the inactive molecules. Second, the reaction volumes 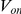 and 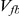 are assumed to be constants, so that the spontaneous-on and feedback rates, 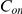 and 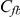 (respectively), are independent of the density of membrane-bound molecules. This may not be a reasonable approximation for regions of high molecule density when the reaction volumes frequently overlap and mass-action kinetics no longer apply. Third, recruitment of inactive molecules to the membrane in our circuit is modeled by simple mass action between active and inactive forms. In reality, positive feedback loops are more complicated and can involve additional molecular components. For example, in budding yeast, active Cdc42 recruits the adaptor protein Bem1, which in turn recruits/activates the Cdc42 GEF (guanine nucleotide exchange factor) Cdc24 [2].

Several biological predictions come out of our work. First, the ability to switch this positive feedback circuit on and off suggests that it could be placed as a primer, upstream of other signaling circuits, to initiate subsequent physiological processes. Such a role has been proposed in the context of Cdc42 polarization in yeast, in which a cytoskeleton-independent positive feedback circuit–with some similar features to the one studied here–acts as a primer for a second actin-dependent positive feedback circuit [2], [36]. Second, our results suggest that over- or under-expression of a reporter probe to monitor a feedback system can have the unintended effect of eliminating the spatial organization that it was intended to observe. Third, our numerical simulations in 2D and 3D suggest that a positive feedback provides a “minimal” model for repressing or initiating molecular aggregation and microdomain formation, such as observed on lipid membranes [1], in the nucleus [50] and in cytosolic puncta [4]. Fourth, our results provide a natural interpretation of (and prediction for) heterogeneity of cells in clustered states by providing a link between numbers of signaling molecules per cell and probabilities of observing off/on states or cluster formation within the population.

Our work points to the intrinsic ability of positive feedback to give rise to spatial clustering. We propose that a positive feedback circuit, operating in the stochastic regime, can create a robust switch that can prevent spurious activation in an “off” state, and can be switched “on” or “off” by simply varying molecular density. As positive feedback loops form a common motif in many signal transduction networks, our work reveals a design principle based on neutral drift dynamics that may lie at the heart of diverse network functions. Additional mechanisms could be coupled to this basic positive feedback module to fine-tune the ability of biological systems to create sharp localized clusters. Finally, the discrete nature of molecular processes means that there can be significant fluctuations from mean behavior described by deterministic models, and stochastic models will be required to capture those effects.

## Materials and Methods

### Analytical results

Derivations of estimates and formulas used in Figures 1–4 are given in the Main Text and Protocol S1.

### Consistency of constants with previous work

In previous work [26], feedback 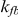 was scaled to maintain a constant fraction of membrane molecules regardless of the total number 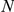 of molecules. The relationship between the previous constants 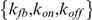 to the current constants 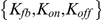 is as follows: 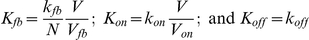.

### Simulations

Simulations in Figure 4 were performed using Matlab version R2009a on a unit as previously described [26]. Parameter values are as shown in Table 3.

All simulations in Figure 5 were performed using the stochastic particle simulator Smoldyn version 2.15. The algorithm for bimolecular reactions in Smoldyn is based on the Smoluchowsky theory of diffusion-limited chemical reactions [51]. We note that the model implemented in Smoldyn differs from the theoretical treatment of the positive feedback circuit in several ways (see Protocol S1, Section 8). Parameter values and Smoldyn code for Figure 5 is given in Protocol S1.

## Supporting Information

Figure S1Polarization frequencies for positive feedback circuits based on two different models of scaling positive feedback. Top panel: positive feedback based entirely on mass action kinetics (current study); Bottom panel: positive feedback normalized to maintain a fixed fraction (set to be 10% in this simulation) of molecules on the membrane at steady state (presented in [26]). Curves and simulations are as in Figure 2A of the main text. Top and bottom panels are averages of 50 or 20 simulations (respectively). We note for the bottom panel that the steady state membrane fraction (black curve) drops below 10% as 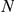 becomes small. This is due to the bimodality of the stationary distribution (see Protocol S1 and [45]); for small 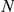, the membrane may be empty frequently. Polarization rates (red curves) also drop for small 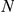. This is due, in part, to the decrease in 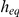. Additionally, regions of the membrane containing ≤20 molecules were not counted as polarized, hence polarization rates may be under-reported. In particular, for the lower panel, fewer than 20 ( = 10%

200) molecules are expected on the membrane when 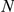
<200, and the low fraction of polarized cells in this regime is in part a reflection of this (arbitrary) cutoff.(PDF)

Figure S2Polarization frequencies for positive feedback with varying spontaneous on-rates. Plots are as in Figures 2A and S1. All simulations performed with 20 replicates; values of 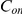 were varied over a 5-fold range (indicated on each panel). Note that even for relatively high values of 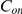, we still observe a sharp boundary below which polarity is not observed.(PDF)

Figure S3Phase plane diagrams for the Smoldyn implementation in (a) cytosolic geometry (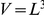), and (b) polar geometry 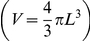, showing the range of 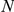 and 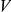 for which polarization will occur. Curves correspond to 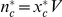 (black curve), 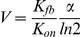, (blue curve), and 
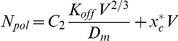
 (red curve). See Protocol S1, Section 8 for detailed derivations of these quantities and Appendix for parameter values used. Abbreviations used: **Off**-homogenous off state, **On**-homogenous on state, **P**-polarity, **MC**-multiple clans.(PDF)

Protocol S1 Supporting Information for “A Density-Dependent Switch Drives Stochastic Clustering and Polarization of Signaling Molecules”, containing details of mathematical derivations.
(PDF)

Video S1 Smoldyn simulation of positive feedback circuit for an increasing number of particles. Repression, emergence, and loss of polarity is observed as the concentration of signaling molecules is increased. See Protocol S1 for code and parameter values.
(MP4)

Video S2 Smoldyn simulation of positive feedback circuit for a fixed number of particles. Recurrent polarity is observed. See Protocol S1 for code and parameter values.
(MP4)
