# Supplementary figures and images for: A Density-Dependent Switch Drives Stochastic Clustering and Polarization of Signaling Molecules
Source: PLoS Comput Biol. 2011 Nov 10;7(11):e1002271. doi: 10.1371/journal.pcbi.1002271 (PMC3213192; doi:10.1371/journal.pcbi.1002271)

Figure S1

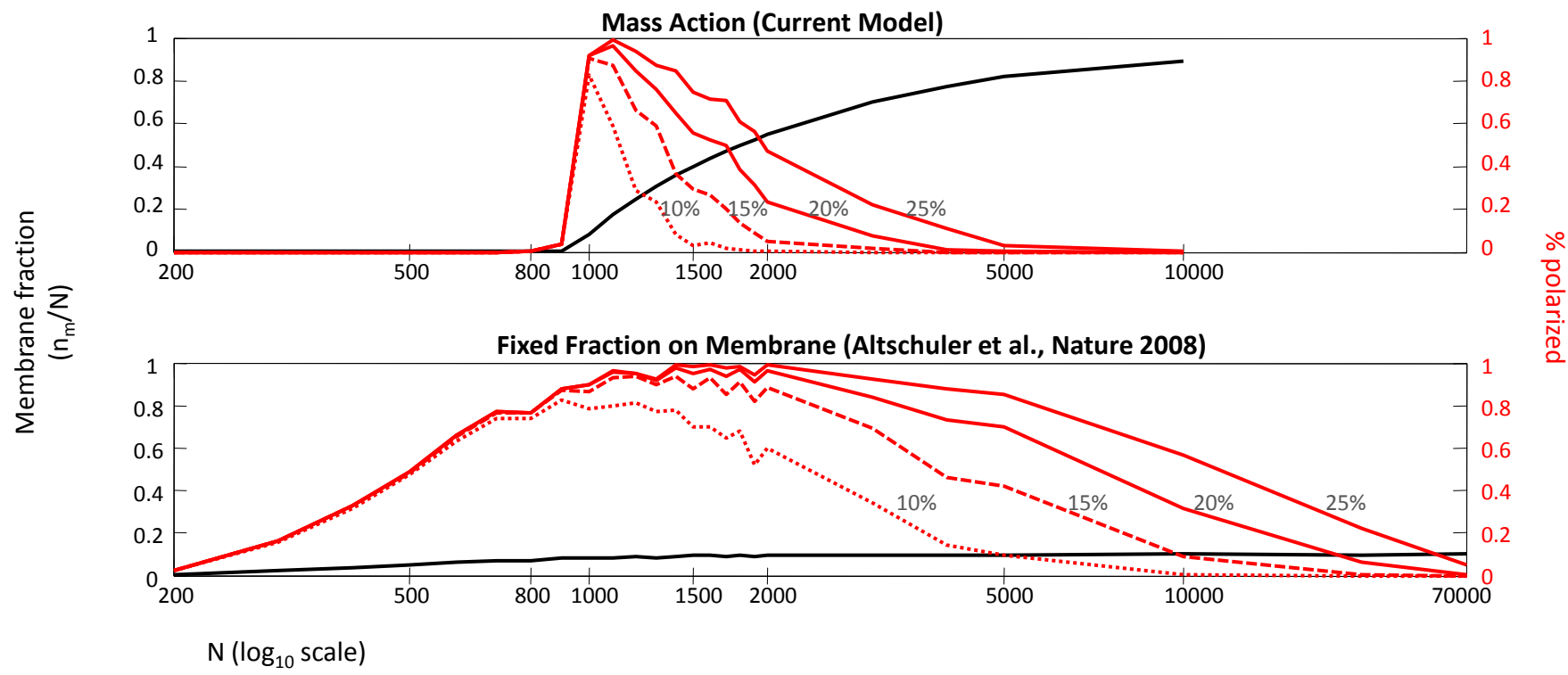

Supplement: Figure S1 — Polarization frequencies for positive feedback circuits based on two different models of scaling positive feedback. Top panel: positive feedback based entirely on mass action kinetics (current study); Bottom panel: positive feedback normalized to maintain a fixed fraction (set to be 10% in this simulation) of molecules on the membrane at steady state (presented in [26]). Curves and simulations are as in Figure 2A of the main text. Top and bottom panels are averages of 50 or 20 simulations (respectively). We note for the bottom panel that the steady state membrane fraction (black curve) drops below 10% as becomes small. This is due to the bimodality of the stationary distribution (see Protocol S1 and [45]); for small , the membrane may be empty frequently. Polarization rates (red curves) also drop for small . This is due, in part, to the decrease in . Additionally, regions of the membrane containing ≤20 molecules were not counted as polarized, hence polarization rates may be under-reported. In particular, for the lower panel, fewer than 20 ( = 10%200) molecules are expected on the membrane when <200, and the low fraction of polarized cells in this regime is in part a reflection of this (arbitrary) cutoff. (PDF) [file pcbi.1002271.s001.pdf]

Figure S2

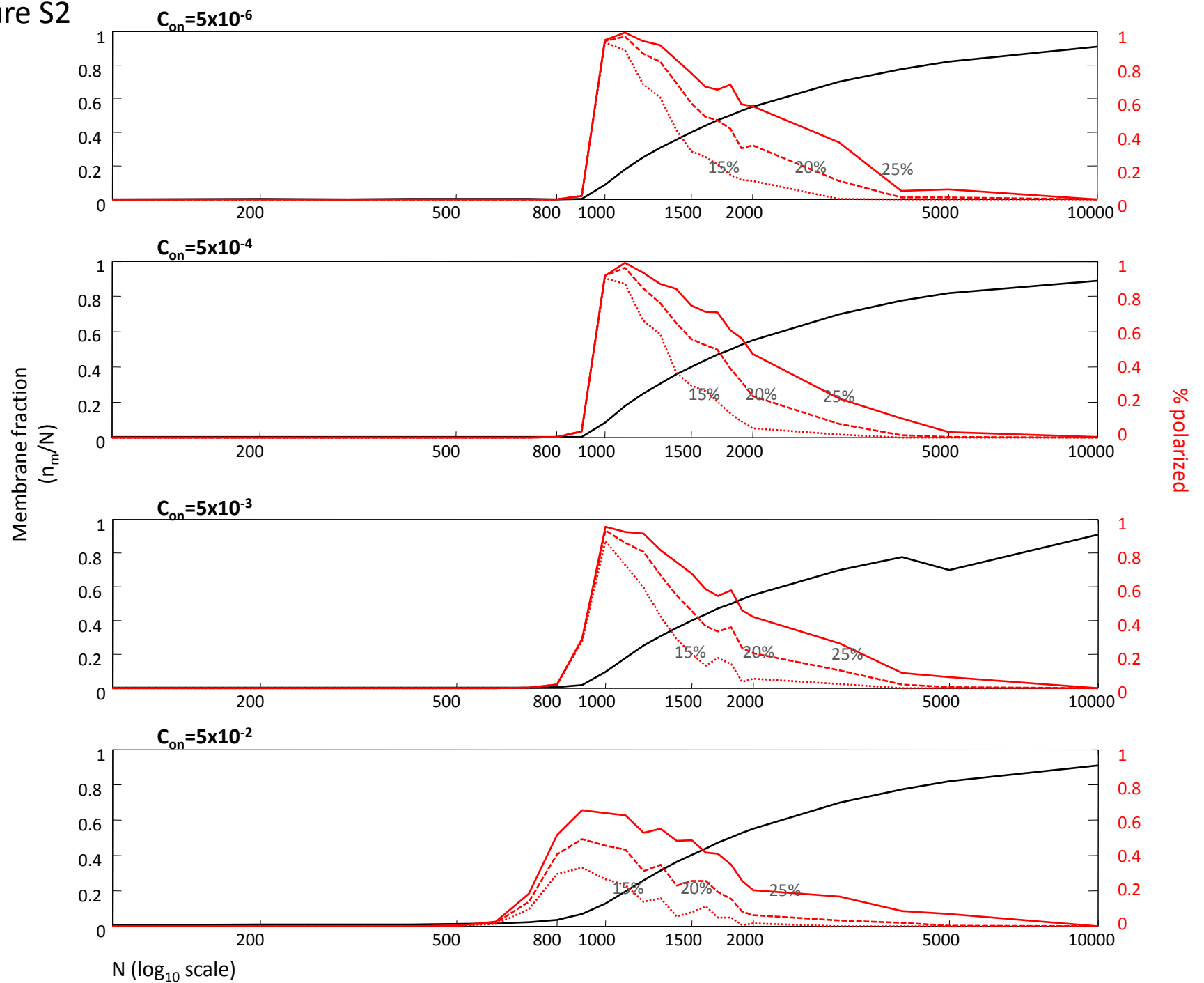

Supplement: Figure S2 — Polarization frequencies for positive feedback with varying spontaneous on-rates. Plots are as in Figures 2A and S1. All simulations performed with 20 replicates; values of were varied over a 5-fold range (indicated on each panel). Note that even for relatively high values of , we still observe a sharp boundary below which polarity is not observed. (PDF) [file pcbi.1002271.s002.pdf]

Figure S3

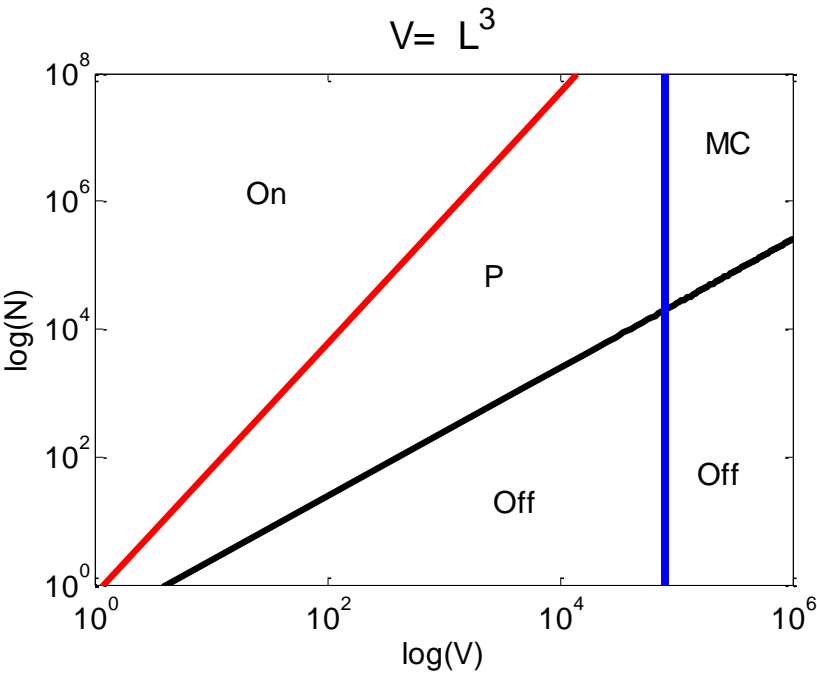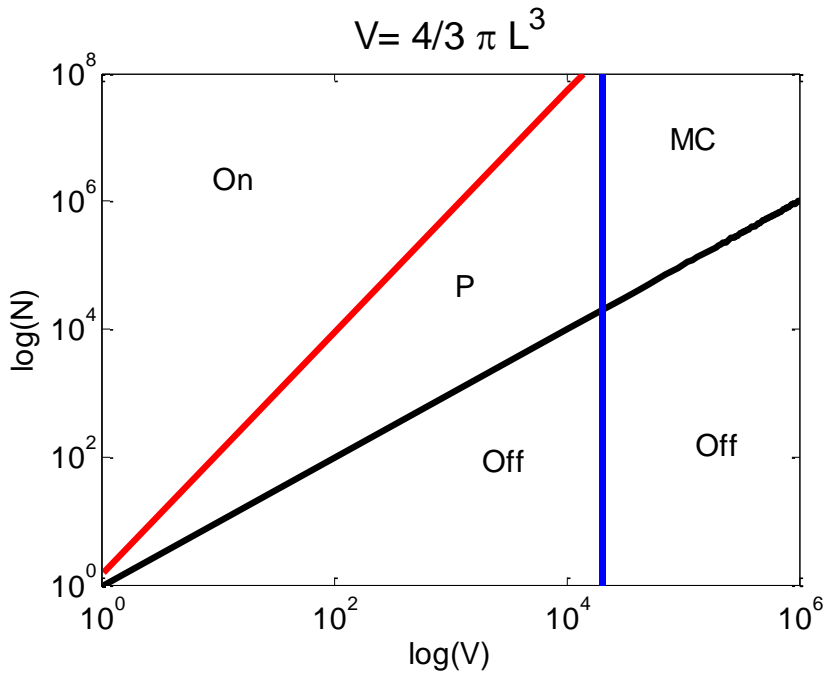

Supplement: Figure S3 — Phase plane diagrams for the Smoldyn implementation in (a) cytosolic geometry (), and (b) polar geometry , showing the range of and for which polarization will occur. Curves correspond to (black curve), , (blue curve), and (red curve). See Protocol S1, Section 8 for detailed derivations of these quantities and Appendix for parameter values used. Abbreviations used: Off-homogenous off state, On-homogenous on state, P-polarity, MC-multiple clans. (PDF) [file pcbi.1002271.s003.pdf]
