# Supplementary material for: A Density-Dependent Switch Drives Stochastic Clustering and Polarization of Signaling Molecules
Source: PLoS Comput Biol. 2011 Nov 10;7(11):e1002271. doi: 10.1371/journal.pcbi.1002271 (PMC3213192; doi:10.1371/journal.pcbi.1002271)
Supplement: Protocol S1 — Supporting Information for “A Density-Dependent Switch Drives Stochastic Clustering and Polarization of Signaling Molecules”, containing details of mathematical derivations. (PDF) [file pcbi.1002271.s004.pdf]

Supplement: Polarization in the simple feedback model

Contents

1. Introduction 1
2. The model 2
3. Dependence of the Parameters on Cell Size and Total Particle Number 2
4. Switching in the Stochastic Model 3
5. The mechanism behind stochastic polarization 8
6. The continuum limit 12
7. Derivation of (17) and competing federations of clans 14
8. The growth of new clans 17
9. Implementation of the Model in Smoldyn 19
Appendix A. Smoldyn Code For a Sphere ($V = \frac{4}{3}\pi L^3$) 21
Appendix B. Smoldyn Code For a 3D Domain ($V = L^3$) 22
Appendix C. Smoldyn Code For a 2D domain ($V = L^2$) 23
References 24

1. Introduction

We previously considered a simple stochastic model for cell polarization in which one chemical species of particles diffuses back and forth between cytosol and membrane of a fixed cell [**1**]. The model, which contained a number of reaction rate constants, made no assumptions on the precise mechanistic nature of the reactions involved in the membrane-cytosol transitions. There, feedback was scaled to maintain a constant fraction of the particles on the membrane as the total number of particles N in the cell varied. We gave an analysis of the behaviour of the system for all possible values of these parameters. In particular we gave an explanation for the polarization which occurs for certain parameter values when N is large. An in-depth mathematical analysis confirming the parameter ranges suggested by numerical simulations in [**1**] was subsequently provided by Gupta [**3**].

In the present paper we modify the model from [**1**], removing the constraint that held the fraction of signaling molecules on the membrane constant. Here all reactions strictly obey mass-action kinetics and, as a consequence, the fraction of membrane-bound particles varies freely as N changes. This alters the behavior of the model: we observe a sharp transition from an "off" state with no molecules on the membrane to a polarized "on" state.

Below, we analyze how this transition from unpolarized to polarized states is affected by the total number N of particles in the cell, and its volume V. Our main conclusion is that for polarization to occur, the particle density N/V in the cell must exceed a certain minimal particle density x_c^* (to be computed below), while the total number of particles N in the cell should lie below a threshold $N_{\max}$ which depends on the reaction rates as well the size of the cell and the rate at which particles on the membrane diffuse (also to be computed below). This allows us to compute a region in the parameter space, where polarization is guaranteed to occur, (main paper, Figure 4C).

In order to understand how the system behavior changes as the total particle number N and cell volume V vary, we recall below relevant arguments from [**1**] that explain how it comes about that the stochastic system can exhibit polarization while the continuum model does not. We next present for our new model arguments that mark the boundaries of the region in parameter space where polarization occurs.

2. The model

The model introduced in the main paper concerns a cell in which molecules move stochastically between cytosol and membrane with the following transition rates:

- Any cytosolic particle can spontaneously move to the membrane with rate $K_{\text{on}} V_{\text{on}}/V$.
- Any membrane-bound particle can move to the cytosol with rate K_{off}.
- Any membrane-bound particle can recruit any cytosolic particle with rate $K_{\text{fb}} V_{\text{fb}}/V$.

Here V is the volume of the cytosol, V_{on} is the volume of the region near the membrane within which spontaneous recruitment to the membrane is possible, and V_{fb} is the volume of the region surrounding a membrane bound particle within which it can recruit a cytosolic particle to the membrane (see the main paper, figure 1B).

In this supplement we will find it convenient to use the constants $C_{\text{off}}, C_{\text{on}}, C_{\text{fb}}$, which are related to $K_{\text{off}}, K_{\text{on}}$, and K_{fb} by

$$C_{\text{off}} = K_{\text{off}}, \quad C_{\text{on}} = K_{\text{on}} \frac{V_{\text{on}}}{V}, \quad C_{\text{fb}} = K_{\text{fb}} \frac{V_{\text{fb}}}{V}. \tag{1}$$

In terms of these parameters the stochastic behavior of the model is defined as follows: if there are N particles in the cell, and if n_c of these are in the cytosol, then during a short time interval of length Δt an on-, off-, or feedback-event occurs with probability

$$C_{\text{on}} n_c \Delta t, \quad C_{\text{off}} (N - n_c) \Delta t, \quad \text{or} \quad C_{\text{fb}} n_c (N - n_c) \Delta t, \quad \text{respectively.}$$

At the same time the membrane-bound particles undergo Brownian motion with Diffusion rate D.

3. Dependence of the Parameters on Cell Size and Total Particle Number

For any fixed choice of parameters $K_{\text{on}}, K_{\text{off}}, K_{\text{fb}}, N, D, V, V_{\text{on}}, V_{\text{off}}$, our present model is the same as the model we considered in [**1**], where we used the following rate constants:

$$k_{\text{on}} = C_{\text{on}}, \quad k_{\text{fb}} = N C_{\text{fb}}, \quad \text{and} \quad k_{\text{off}} = C_{\text{off}}.$$

The difference with [**1**] lies in how the reaction rates change with the size of the cell and the total number of molecules it contains. In [**1**] the constants $k_{\text{on}}, k_{\text{off}}, k_{\text{fb}}$ were kept constant, while here, following mass-action kinetics, the constants $K_{\text{on}}, K_{\text{off}}, K_{\text{fb}}$ are fixed.

It follows from (1) that the constants $C_{\text{on}}, C_{\text{off}}, C_{\text{fb}}$ also do not change with N, but they do depend on the size of the cell. If one considers cells of varying volumes V, then the spontaneous recruitment volume V_{on} must also vary. Under the most natural hypothesis V_{on} should be proportional to the area of the cell membrane, and assuming the cells are spherical, the "on-volume" V_{on} should therefore vary proportionally to $V^{2/3}$.

All rates $C_{\text{on}}, C_{\text{off}}, C_{\text{fb}}, k_{\text{on}}, k_{\text{off}}, k_{\text{fb}}, K_{\text{on}}, K_{\text{off}}$, and K_{fb} have units $[\texttt{time}]^{-1}$.

4. Switching in the Stochastic Model

In the stochastic model the total number $n_c(t)$ of particles on the membrane evolves by a continuous time Markov process, whose master equation describes the time evolution of the probability distribution of $n(t)$. We derive the stationary distribution for the number of particles $n_c(t)$ in the cytosol (as in [**1**]), and analyze how it changes when N decreases from $N > n_c^*$ to $N < n_c^*$, where n_c^* is the critical particle number

$$n_c^* = \frac{C_{\text{off}}}{C_{\text{fb}}} = \frac{K_{\text{off}}}{K_{\text{fb}}} \frac{V}{V_{\text{fb}}}. \tag{2}$$

The stationary distribution is the time independent solution to the master equation governing the $n_c(t)$ process. If $\{p_n : 0 \le n \le N\}$ is the stationary distribution, then p_n is the average amount of time the cytosol population of a particular cell will be $n_c(t) = n$, i.e. the average amount of time the cell will have exactly n particles in its cytosol.

4.1. The case $C_{\text{on}} = 0$. If $C_{\text{on}} = 0$ then the stationary distribution is concentrated at $n_c = N$. Indeed, if all particles are in the cytosol, and if $C_{\text{on}} = 0$, then there is no way particles can move to the membrane. In the long run the system will always end up in this state, and once in the state $n_c(t) = N$ the system must remain at $n_c(t) = N$. Therefore, over a sufficiently long time interval, the fraction of time the system spends in the state $n_c(t) = N$ approaches 1. This, by definition, implies that the stationary distribution is given by

$$p_n = 0 \text{ for } n = 0, 1, \ldots, N-1, \text{ and } p_N = 1. \tag{3}$$

4.2. The general formula for p_n when $C_{\text{on}} > 0$. The stochastic variable $n_c(t)$ evolves by discrete jumps of ± 1, and one can always find explicit expressions for the stationary distributions of such systems (e.g. see [**5**, Ch.VI] or [**4**, §6.11]).

The rates at which the jumps occur are

$$\begin{aligned} (n-1) \to n \quad &: \quad C_{\text{off}}(N - n + 1) \\ n \to (n-1) \quad &: \quad C_{\text{fb}}(N-n)n + C_{\text{on}} n \end{aligned}$$

If we write $p_n(t) = \Pr[n_c(t) = n]$, then the master equation can be written as

$$\frac{dp_n}{dt} = W_{n+1} - W_n, \tag{4}$$

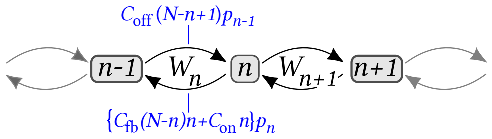

Figure 1. The random walk for the number of particles $n_c(t)$ in the cytosol. W_n is the net rate at which probability flows from state n to state $n-1$. In equilibrium this rate vanishes (see §4.2).

for $n = 1, 2, \ldots, N-1$, and

$$\frac{dp_0}{dt} = +W_1, \qquad \frac{dp_N}{dt} = -W_N.$$

Here

$$W_n = [C_{\rm fb}(N-n)n + C_{\rm on}n]\,p_n - C_{\rm off}(N-n+1)p_{n-1}$$

is the rate at which probability flows from the state $n_c(t) = n$ to the state $n_c(t) = n-1$ (see figure 1). For the stationary distribution one has $W_{n+1} = W_n$ for all $n = 1, 2, \ldots, N-1$ and also $W_1 = W_N = 0$. This implies $W_n = 0$ for all n, and thus

$$\text{(5)} \qquad \frac{(N-n)p_n}{(N-n+1)p_{n-1}} = \frac{1}{n}\frac{C_{\rm off}}{C_{\rm fb} + \frac{C_{\rm on}}{N-n}} = \frac{1}{n}\frac{n_c^*}{1 + \frac{C_{\rm on}/C_{\rm fb}}{N-n}} = \frac{n_c^*}{n}\Big(1 + \frac{\gamma}{N-n}\Big)^{-1},$$

in which

$$\text{(6)} \qquad \gamma = \frac{C_{\rm on}}{C_{\rm fb}} = \frac{K_{\rm on}V_{\rm on}}{K_{\rm fb}V_{\rm fb}},$$

is the non-dimensionalized on-rate, and n_c^* is given by (2). From here one finds for $n = 0, 1, 2, 3, \cdots, N-1$ that

$$\frac{p_n}{p_0} = \frac{N}{N-n}\frac{(n_c^*)^n}{n!}\prod_{k=1}^{n-1}\Big(1 + \frac{\gamma}{N-k}\Big)^{-1}.$$

To get p_N we use that $W_N = 0$ implies

$$\text{(7)} \qquad \frac{p_N}{p_{N-1}} = \frac{C_{\rm off}}{C_{\rm on}N} = \frac{n_c^*}{N\gamma},$$

which leads to

$$\frac{p_N}{p_0} = \frac{N}{\gamma}\frac{(n_c^*)^N}{N!}\prod_{k=1}^{N-2}\Big(1 + \frac{\gamma}{N-k}\Big)^{-1}.$$

4.3. Approximation for $C_{\rm on} \ll C_{\rm fb}$. If we now assume that $C_{\rm on}$ is much smaller than $C_{\rm fb}$, so that $\gamma \ll 1$, then we find the following approximation for the

logarithm of the product which appears in our expressions for p_n/p_0,

$$\begin{aligned}\ln\prod_{k=1}^{n-1}\Big\{1+\frac{\gamma}{N-k}\Big\}^{-1} &= -\sum_{k=1}^{n-1}\ln\Big\{1+\frac{\gamma}{N-k}\Big\}\\ &= -\sum_{k=1}^{n-1}\Big\{\frac{\gamma}{N-k}+O\big(\frac{\gamma^2}{(N-k)^2}\big)\Big\}\\ &= -\Big\{\sum_{k=1}^{n-1}\frac{\gamma}{N-k}\Big\}+O(\gamma^2)\\ &= -\gamma\ln\frac{N}{N-n}+O(\gamma).\end{aligned}$$

Thus

$$\prod_{k=1}^{n-1}\Big\{1+\frac{\gamma}{N-k}\Big\}^{-1}=\Big(\frac{N}{N-n}\Big)^{-\gamma}e^{O(\gamma)}. \tag{8}$$

Here $O(\gamma)$ is a quantity which is bounded by $C\gamma$, where C is a constant which does not depend on N, n, n_c^*. Applying this to our explicit expressions for p_n/p_0 we get

$$\frac{p_n}{p_0}=\frac{(n_c^*)^n}{n!}\Big(\frac{N}{N-n}\Big)^{1-\gamma}e^{O(\gamma)} \text{ for } n=0,1,2,\dots,N-1 \tag{9}$$

and

$$\frac{p_N}{p_0}=\frac{1}{\gamma}\frac{(n_c^*)^N}{N!}N^{1-\gamma}e^{O(\gamma)}. \tag{10}$$

The ratios (9) and (10), combined with the requirement that the probabilities p_n must add up to 1, completely determine the p_n.

4.4. The case $n_c^* < N$; bimodality for small γ. The first factor $(n_c^*)^n/n!$ in (9) corresponds to an unnormalized Poisson distribution with parameter n_c^*. As a function of n, the quantity $(n_c^*)^n/n!$ reaches its maximum at $n = n_c^*$, and, compared to this maximum it is negligible when $|n - n_c^*| \gg \sqrt{n_c^*}$. Thus, if we ignore p_N as given by (10), then we find that the stationary distribution is concentrated near the equilibrium value n_c^* predicted by the deterministic model. When γ is large it follows from (9) and (10) that one can indeed ignore p_N, but when γ is small one cannot. Indeed, in the extreme case where $\gamma = 0$, (10) requires $p_N = 1$ and $p_i = 0$ for all $i = 0, 1, \dots, N-1$. This is consistent with the description of the stochastic model for $C_{\mathrm{on}} = 0$ given above in §4.1.

When γ is very small, but still positive, the state $n_c(t) = N$ is not absorbing. However, if the system ever ends up in this state, it can only leave this state through an on-event. The rate at which these occur is $C_{\mathrm{on}}n_c(t) = C_{\mathrm{on}}N = \gamma C_{\mathrm{fb}}N$, and thus the average time the system will spend in the state $n_c(t) = N$ is $(C_{\mathrm{fb}}N\gamma)^{-1} \sim \gamma^{-1}$. This time can be very long when γ is small.

We can now describe the general dynamic behavior of the system. Most of the time the system is either in the state $n_c(t) = N$ (membrane empty), or in the state where it fluctuates at $n_c(t) = n_c^* + O(\sqrt{n_c^*})$. The time it takes the system to leave the empty-membrane state $n_c(t) = N$, is proportional to γ^{-1}. When the system leaves this state it evolves back to the stable equilibrium region $n_c(t) \approx n_c^*$ with high probability. Subsequently $n_c(t)$ will fluctuate around n_c^* until a sufficiently large fluctuation drives $n_c(t)$ back to the empty membrane value $n_c(t) = N$.

The recurrent back and forth cycling of the system between the two states $n_c(t) = N$ and $n_c(t) \approx n_c^*$ is reflected in the possible bimodal profile of the stationary distribution (main paper, figure 3D, inset). Depending on γ, the stationary distribution will have one peak at $n = N$ and another Gaussian-shaped peak near $n = n_c^*$ with width $\sim \sqrt{n_c^*}$. The relative heights of these two peaks is indicative of the average time it takes the system to leave either of the corresponding states.

This bimodal profile of the stationary distribution only appears when the parameter γ lies in a critical range. When γ is too small the membrane-empty state is so close to being perfectly absorbing that the stationary distribution will only have a peak at $n = N$. If, on the other hand, γ is too large then only the peak at $n \approx n_c^*$ will dominate.

The range of γ at which bimodality occurs will depend on N. In order to determine this range, we compute the ratio

$$p_N : (p_0 + \cdots + p_{N-1}),$$

and see where the two probabilities are comparable.

To compute the sum $p_0 + \cdots + p_{N-1}$ we note that when $N > n_c^*$ the Poisson distribution $(n_c^*)^n/n!$ reaches its maximum in the interval $0 \le n < N$, and one therefore has

$$\sum_{n=0}^{N-1} \frac{(n_c^*)^n}{n!} = e^{n_c^*} - \sum_{n \ge N} \frac{(n_c^*)^n}{n!} = (1 + o(1))e^{n_c^*}.$$

Here $o(1)$ is a quantity which tends to zero as $N \to \infty$, assuming that the ratio N/n_c^* stays larger than 1 (this will certainly be the case if, as in this paper, n_c^* stays fixed, and $N \to \infty$; however it also holds in the scaling of [**1**] where the ratio N/n_c^* was kept fixed at some value $1/h_{\mathrm{eq}}$). Applying this to (9) we get

$$\frac{p_0 + \cdots + p_{N-1}}{p_0} = e^{n_c^*} \left(\frac{N}{N - n_c^*} \right)^{1-\gamma} e^{O(\gamma)} (1 + o(1)).$$

Dividing this into (10) we get

$$\frac{p_N}{p_0 + \cdots + p_{N-1}} = \frac{1}{\gamma} \frac{(n_c^*)^N e^{-n_c^*}}{N!} (N - n_c^*)^{1-\gamma} \big(1 + o(1) + O(\gamma)\big). \tag{11}$$

Hence $p_N \approx p_0 + \cdots + p_{N-1}$ will hold if

$$\gamma (N - n_c^*)^\gamma \approx \frac{(n_c^*)^N e^{-n_c^*}}{N!} (N - n_c^*). \tag{12}$$

If n_c^* is fixed, then the Right Hand Side decreases faster than exponentially as $N \to \infty$ (due to the $N!$). Even if one allows n_c^* to vary with N with $n_c^* \le \theta N$ for some constant $\theta < 1$, one still has

$$\begin{aligned} \frac{(n_c^*)^N e^{-n_c^*}}{N!} (N - n_c^*) &\approx \frac{(\theta N)^N e^{-\theta N}}{N!} (N - n_c^*) \\ &\le \frac{(\theta N)^N e^{-\theta N}}{\sqrt{2\pi N} N^N e^{-N}} N \\ &\approx \big(\theta e^{1-\theta}\big)^N \sqrt{N/2\pi}, \end{aligned}$$

by Stirling's formula. Since $\theta e^{1-\theta} < 1$ for all $\theta < 1$ the RHS in (12) decreases exponentially with N. Solving (12) for γ we find that $(N - n_c^*)^\gamma \approx 1 + \gamma \ln(N - n_c^*)$ can be omitted, while the factor $\sqrt{N}$ is also negligible on a logarithmic scale

compared to the exponential factor $(\theta e^{1-\theta})^N$. We therefore find that the stationary distribution will be bimodal, in the sense that it has two peaks of equal probability, when

$$\gamma \approx \gamma_{\mathrm{bm}} \stackrel{\mathrm{def}}{=} \left(\theta e^{1-\theta}\right)^{N+o(N)}, \quad \theta = \frac{n_c^*}{N}. \tag{13}$$

If N is fixed then it follows from (11) that changing γ by a factor of 10 will result in a tenfold change in the ratio between the probabilities contained in the two peaks. Bimodality will therefore mostly be visible in the range

$$\frac{1}{10}\gamma_{\mathrm{bm}} < \gamma < 10\gamma_{\mathrm{bm}}$$

This range, in which bimodality occurs, varies strongly with N. E.g. if n_c^* is at most $N/2$, then $\theta \le 0.5$ and $\theta e^{1-\theta} \approx 0.824\dots$. Every increase of N by 20 decreases γ_{bm} by a factor $(\theta e^{1-\theta})^{20} \approx 0.02 \approx \frac{1}{50}$. If $n_c^* \le \frac{1}{10}N$, so that $\theta \le 0.1$, then $\theta e^{1-\theta} \approx 0.246$ and every increase of N by five particles decreases γ_{bm} by a factor $0.0009 \approx 10^{-3}$.

4.5. The case $N < n_c^*$. When $N < n_c^*$, it follows from (5) that

$$\frac{p_n}{p_{n-1}} = \frac{1 + \frac{1}{N-n}}{1 + \frac{\gamma}{N-n}} \cdot \frac{n_c^*}{n} \text{ for all } n < N.$$

If $\gamma < 1$ then we see that $\frac{p_n}{p_{n-1}} > \frac{n_c^*}{N} > 1$. We also found in (7) that

$$p_N = \frac{n_c^*}{N\gamma} p_{N-1} > p_{N-1}.$$

Hence the probabilities p_n in the stationary distribution are strictly increasing in $n = 0, 1, 2, \cdots, N$. They increase exponentially and the last probability, p_N, is a factor $O(\gamma^{-1})$ larger than the one before last, p_{N-1}.

It follows that for $k > 0$ one has

$$p_{N-k} \le \gamma \Big(\frac{N}{n_c^*}\Big)^k p_N$$

and hence

$$p_0 + \cdots + p_{N-1} \le \gamma \frac{n_c^*}{n_c^* - N}\, p_N.$$

Therefore in the stationary distribution the probability that the cytosol population is maximal, i.e. $n_c(t) = N$ is $1 - O(\gamma)$. Moreover, in the case that there actually are particles on the membrane (which happens with probability only $O(\gamma)$), the conditional expectation of the number of particles on the membrane is

$$\mathbf{E}\big[N - n_c(t) \mid n_c(t) < N\big] \le \frac{1}{1 - \frac{N}{n_c^*}} = \frac{n_c^*}{n_c^* - N}.$$

For instance, when $N < \frac{1}{2}n_c^*$, this expected number of particles is two: in this case the probability that there are particles on the membrane is very small, namely $O(\gamma)$, and even when there are particles on the membrane the expected number of particles is two.

4.6. Summary. Both the ODE model in the main paper and the analysis of the stochastic counterpart above show that, when C_{on} is much smaller than the other two rate constants C_{off} and C_{fb}, the total number of particles in the cytosol will settle to an equilibrium given by either n_c^* or N, depending on which is smaller. Thus, as N increases, a transition occurs at $N = n_c^*$: for $N < n_c^*$ all particles will remain in the cytosol (and no polarization can occur), while for $N > n_c^*$ a fixed number (n_c^* with fluctuations of order $\sqrt{n_c^*}$) of particles remains in the cytosol with all other particles moving to the membrane.

The critical density

$$x_c^* = \frac{n_c^*}{V} = \frac{K_{\text{off}}}{K_{\text{fb}} V_{\text{fb}}} \tag{14}$$

only depends on the mass-action constants $K_{\text{off}}, K_{\text{fb}}, V_{\text{fb}}$ and, in particular, does not depend of N or V.

5. The mechanism behind stochastic polarization

5.1. Clans and polarization. We have seen that polarization will not occur when $N < n_c^*$, simply because no or almost no particles are on the membrane for this range of N. From here on we assume that $N > n_c^*$, and we recall how polarization, when it occurs, can be explained by considering the genealogy of particles as they move back and forth between membrane and cytosol in between "on-events."

Divide the membrane into a number of regions, and, at time $t = 0$ split the population of membrane-bound particles into "clans," assigning one clan to each region. Thus at time $t = 0$ the particles which happen to be in the same region form a clan, and different regions host different clans. Thereafter, for $t > 0$, each particle that moves to the membrane through an on-event starts an entirely new clan of its own, while a particle that is recruited to the membrane receives the clan identity of the recruiting particle (its "parent"). When a particle leaves the membrane it loses its clan identity. As the membrane-bound particles diffuse on the membrane, they wander away from the original region that contained their ancestors.

In between on-events no new clans are ever formed while existing clans grow and shrink in population. Since the total membrane population remains more or less constant at the expected value $N - n_c^*$ (with standard deviation $O(\sqrt{n_c^*})$), the clans are competing with each other for the membrane-bound particles. The rate at which any given clan will gain or lose a particle is proportional to its size, namely, if a clan has m particles, then the probability that it will recruit a new particle during a time interval of length Δt is

$$C_{\text{fb}} \times m \times n_c(t) \times \Delta t,$$

while the probability that it will lose a particle is

$$C_{\text{off}} \times m \times \Delta t.$$

Since the individuals in all clans have the same "death/reproduction probabilities," all clans are equally fit and the relative ratios of the clan sizes will undergo a neutral drift during which clans occasionally die out. In the end only one clan will survive.

To see how polarization comes about consider the locations of the particles in the sole surviving clan. While particles were cycling back and forth between membrane and cytosol, particles on the membrane were diffusing and some will have moved away from the region in which they originally found themselves. If, by

the time only one clan has survived, one can guarantee that its clan members will not have diffused far away from the clan's original region, then *all* membrane bound particles will be located near one of the small regions, and thus the membrane-bound particles will form a localized cluster on the membrane.

For polarization to occur two conditions must therefore be met. First, the frequency of on-events must be so low that there is enough time before their contribution to the membrane-bound particle population becomes significant to allow all clans but one to become extinct with high probability. Second, the diffusion rate of membrane-bound particles must be so low that, while the number of clans is being reduced to one, the average distance particles diffuse away from their original ancestor is small compared to the radius of the cell.

In these arguments we analyze the distribution of membrane bound particles assuming no on-events occur. Let us now consider the effect of on-events. At each on-event a new clan is created. The population of this new clan is 1, while the total population of all the already present clans is roughly $N - n_c^* \approx N$ for large N. The prospects for survival of the new clan are small, and its most likely fate is a rapid extinction. In this case we may ignore the brief appearance of this small new clan in our analysis of the distribution of the original particles and their descendants on the membrane. To conclude that polarization will occur we therefore can relax our assumption that no on-events occur to the assumption that the descendants of particles which arrive on the membrane through on-events never make up more than a small percentage of the total membrane population.

5.2. The range of N for which polarization will occur. Here we estimate the time between on-events, how long it takes for almost all clans to become extinct, and how far particles can diffuse in that time. If there are n_c^* particles in the cytosol, then on-events occur at a rate of $C_{\text{on}} n_c^*$ per second, and thus the expectation of the time between on-events is of the order

$$T_{\text{on}} = \left(n_c^* C_{\text{on}}\right)^{-1} = \frac{C_{\text{fb}}}{C_{\text{on}} C_{\text{off}}} = \frac{K_{\text{fb}} V_{\text{fb}}}{K_{\text{on}} K_{\text{off}} V_{\text{on}}}. \tag{15}$$

This time T_{on} does not depend on N. Its only dependence on the size of the cell is through V_{on}. If we assume that the cell is spherical, then V_{on} is proportional to the surface area of the membrane, so that $V_{\text{on}} \sim V^{2/3}$ and $T_{\text{on}} \sim V^{-2/3}$.

Looking beyond the first on-event one can keep track of all particles which arrived through an on-event and their descendants, and one can then estimate how long it will take before these particles make up more than a certain fraction α of the total membrane population. In §8 we find that for small α (e.g. $\alpha \le 10\%$) the expected time is

$$T_\alpha = \alpha(N - n_c^*) T_{\text{on}}. \tag{16}$$

We now consider the particles which originally were on the membrane and their descendants. If at any moment one divides the membrane-bound particles into clans, and only considers the descendants of these original clans, then, by definition, the number of clans cannot increase. We will argue in §7 that the expected time within which the number of surviving original clans is halved is given by

$$T_{\text{half}} = \frac{N - n_c^*}{C_{\text{off}}} \ln 2. \tag{17}$$

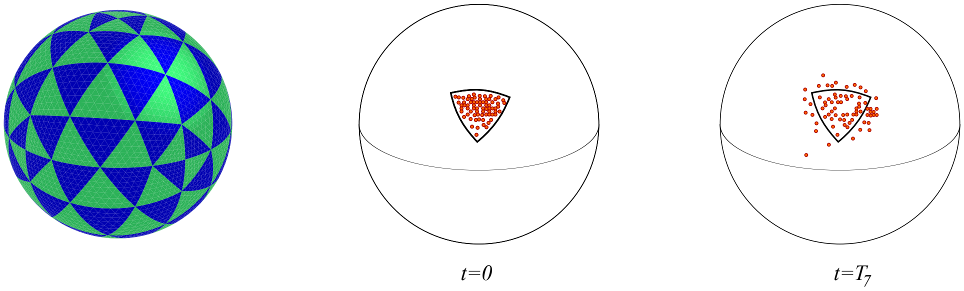

Figure 2. Left: We divide the sphere into 128 triangles. **Middle:** at $t = 0$ the particles inhabiting one of the triangles form a clan. This way, a clan is associated to each triangle on the sphere. Only one of the 128 clans is shown. **Right:** at time T_7 the only remaining particles on the membrane all belong to the same clan. By definition, these clan particles trace their ancestry to one of the 128 triangles. At time T_7 they will still be close to this ancestral triangle, provided the membrane diffusion rate D is not too large.

Thus if one starts with 2^k clans, then the expected time at which only one clan is left (assuming no on-events occur) is

$$T_k = k \cdot T_{\text{half}} = k \frac{N - n_c^*}{C_{\text{off}}} \ln 2 = \frac{N - n_c^*}{C_{\text{off}}} \ln 2^k.$$

Within this time interval the expected distance by which the particles in the only surviving clan will have moved is[1]

$$d = 2\sqrt{DT_k}.$$

If the original 2^k clans were obtained by dividing the membrane into 2^k pieces of equal size, then the remaining clan at time T_k will be within a neighborhood of size d surrounding the region containing its original ancestors.

We now choose a specific subdivision of the membrane into small regions by subdividing it into many small triangles. One can construct such a triangulation of the membrane by beginning with a coarse triangulation (e.g. if the membrane is a sphere, project an octahedron onto the sphere) and then repeatedly splitting each triangle into four smaller triangles by cutting each edge in two, and connecting the midpoints. If the original coarse triangulation had eight triangles, then repeating this procedure m times leads to a triangulation of the membrane into $8 \times 4^m = 2^{2m+3}$ equal triangles. The area of each triangle is $A_m = 2^{-2m-3}A$, where A is the area of the membrane ($A = 4\pi R^2$ if the membrane is a sphere of radius R). Assuming that the triangles are equilateral the radii r_m of their circumscribed circles satisfy $A_m = \frac{3}{4}\sqrt{3}r_m^2$, and therefore the radius r_m is given by

$$r_m = \sqrt{\frac{2^{-2m-3}A}{\frac{3}{4}\sqrt{3}}} = 2^{-m}\sqrt{\frac{2\pi}{3\sqrt{3}}}R. \tag{18}$$

[1]This expression for the distance travelled takes into account that the diffusion is occurring on a two dimensional membrane. If the particles were diffusing in a three dimensional region rather than on a two dimensional membrane then we would have $d = \sqrt{2\nu DT_k}$ with $\nu = 3$.

If the membrane is spherical, then the radius of the upper hemisphere (measured along the surface) on the membrane is $\frac{\pi}{2}R$.

We choose $m = 2$, so that we have divided the membrane into $2^7 = 128$ triangles. The ratio between the radius of one of these triangles and the radius of a hemisphere is

$$\frac{r_2}{\pi R/2} = \frac{\frac{1}{4}\sqrt{2\pi/3\sqrt{3}}\,R}{\pi R/2} = \frac{1}{\sqrt{6\pi\sqrt{3}}} \approx 0.175\ldots.$$

Since we start with 2^7 clans, the expected time until only one of these survives is

$$T_7 = 7\ln 2 \times \frac{N - n_c^*}{C_{\text{off}}}.$$

this leads us to the first of two sufficient conditions which together guarantee that polarization will occur. Namely, the descendants of particles which came to the membrane in an on-event cannot make up more than a small fraction α of the total membrane population at any time $t < T_7$. Thus we are led to the requirement $T_7 \ll T_\alpha$, i.e.

$$\alpha(N - n_c^*)\frac{C_{\text{fb}}}{C_{\text{on}}C_{\text{off}}} \gg 7\ln 2 \times \frac{N - n_c^*}{C_{\text{off}}},$$

i.e.

$$\frac{C_{\text{on}}}{C_{\text{fb}}} \ll \frac{\alpha}{7\ln 2}(\approx 0.02 \text{ if } \alpha = 0.1). \tag{19}$$

We note that this condition does not depend on the total particle number N. Note, however that it does depend on V indirectly, as

$$\frac{C_{\text{on}}}{C_{\text{fb}}} = \frac{K_{\text{on}}V_{\text{on}}}{K_{\text{fb}}V_{\text{fb}}}.$$

If, as before, we assume that V_{fb} is independent of V and $V_{\text{on}} \approx V^{2/3}$, then (19) becomes

$$V \ll \left(\frac{\alpha K_{\text{fb}}V_{\text{fb}}}{7\ln 2 K_{\text{on}}}\right)^{3/2}.$$

The second of the two sufficient conditions imposes a limitation on the diffusion rate. In the time T_7 it takes for all but one clan to die out, clan members will have wandered a distance

$$d = 2\sqrt{DT_7} = 2\sqrt{7\ln 2}\sqrt{D(N - n_c^*)/C_{\text{off}}}$$

from their ancestral triangle. If we assume that $d \leq r_2$, then the particles of the surviving clan will be located in a region of radius $2 \times r_2 \approx 0.35 \times \frac{\pi}{2}R$, i.e. about one third of the radius of a hemisphere: in this case the cell is polarized.

This second condition for polarization is therefore met if

$$d \leq r_2 \iff 2\sqrt{7\ln 2}\sqrt{D(N - n_c^*)/C_{\text{off}}} \leq \tfrac{1}{4}\sqrt{2\pi/3\sqrt{3}}R,$$

which is equivalent to

$$N - n_c^* \leq \frac{\pi}{24\sqrt{3} \times 28\ln 2}\frac{C_{\text{off}}R^2}{D} \approx 0.039 \times \frac{C_{\text{off}}R^2}{D}.$$

In terms of the volume $V = \frac{4}{3}\pi R^3$ of the cell this restriction is

$$N \le N_{\mathrm{pol}} \stackrel{\mathrm{def}}{=} \frac{0.039}{(4\pi/3)^{2/3}} \times \frac{C_{\mathrm{off}} V^{2/3}}{D} + n_c^* \approx 0.015 \times \frac{K_{\mathrm{off}}}{D} V^{2/3} + \frac{K_{\mathrm{off}}}{K_{\mathrm{fb}} V_{\mathrm{fb}}} V. \tag{20}$$

Note that N_{pol} is always greater than the critical number needed for polarity n_c^*. Thus if $C_{\mathrm{on}}/C_{\mathrm{fb}} \ll 0.02$ and $N \le N_{\mathrm{pol}}$ both hold, then polarization will occur. If the second of these conditions is not met, so that $N \gg N_{\mathrm{pol}}$, then before on-events have claimed more than 10% of the membrane population, a single surviving clan will appear, but within the time it takes this clan to emerge its member particles will have spread out over the entire cell membrane.

6. The continuum limit

If there are many particles on the membrane then one can try to model their distribution on the membrane in terms of a continuous particle density $u(x, t)$ depending on space and time. Combined with this assumption, the model then leads to a reaction diffusion equation for u. Here we derive this equation. The arguments in [**1**] show that polarization in this continuum model, if it occurs at all, only occurs for suitable initial data, and then is only short lived.

Below we derive differential equations for quantities which, strictly speaking, are integer valued. In doing this we adopt the usual interpretation of these differential equations. Namely, when the number of particles is large we *assume* that any random variable (integer valued or not) is given by a continuous real valued function of time plus a small noise term. The main, continuous, term is deterministic and satisfies a differential equation, while all the stochasticity of the model is contained in the small "noise term." The assumption that the noise term can be ignored, as well as the related assumption that the particle distribution on the membrane is described by a continuous density are not always valid. The arguments about clans tell us the parameter range in which the continuum approximation fails.

6.1. Derivation of the Reaction Diffusion Equation. Consider a small piece A of the membrane, and let $|A|$ and $|M|$ denote the areas of this piece A and of the entire membrane.

We let $u(x, t)$ denote the density at time t of membrane bound particles. Thus the number of particles in the region A of the membrane is

$$n_A(t) = \int_A u(x, t)\,dx.$$

The rate at which the number of particles in A changes due to the reactions is

$$\frac{dn_A}{dt} = C_{\mathrm{on}} n_c \frac{|A|}{|M|} + C_{\mathrm{fb}} n_c n_A - C_{\mathrm{off}} n_A.$$

Dividing by $|A|$ and letting the region A shrink to a point on the membrane gives us the reaction terms governing the evolution of the density u

$$\frac{du}{dt} = \frac{C_{\mathrm{on}}}{|M|} n_c + C_{\mathrm{fb}} n_c u - C_{\mathrm{off}} u.$$

Adding diffusion gives us the Reaction Diffusion Equation for u

$$\frac{\partial u}{\partial t} = \tfrac{1}{2} D \Delta u + \frac{C_{\mathrm{on}}}{|M|} n_c + C_{\mathrm{fb}} n_c u - C_{\mathrm{off}} u. \tag{21}$$

The particle density must satisfy

$$n_c(t) + \int_M u(x,t)dx = N.$$

Together with (21) this implies an ODE for the number of particles in the cytosol, namely

$$\frac{dn_c}{dt} = -\int_M u dx = -C_{\mathrm{on}} n_c - C_{\mathrm{fb}}(N - n_c)n_c + C_{\mathrm{off}}(N - n_c). \tag{22}$$

6.2. The vanishing of spatial patterns. The equation (21) satisfied by the membrane particle density $u(x,t)$ is linear in u, and, consequently, diffusion will erase any spatial variations in this density. To make a more precise statement consider the average density $\bar{u}(t)$ given by

$$\bar{u}(t) = \frac{1}{|M|}\int_M u(x,t)\, dx,$$

and the *mean square deviation from the average* defined by

$$\sigma(t)^2 = \frac{1}{|M|}\int_M \left(\frac{u(x,t) - \bar{u}(t)}{\bar{u}(t)}\right)^2\, dx. \tag{23}$$

If $\sigma(t)^2 = 0$ then the density $u(x,t)$ must everywhere be equal to its average, and thus be spatially constant; if $\sigma(t)^2$ is small, then the density u will be close to its spatial average , and thus it will be nearly constant. Thus the relevance of the quantity $\sigma(t)^2$ is that it measures the size of any spatial features the particle density $u(x,t)$ may have.

Theorem. *The mean square deviation $\sigma(t)^2$ decays exponentially. If λ_1 is the first eigenvalue of the Laplace operator Δ of the membrane, then one has*

$$\sigma(t)^2 \le e^{-\lambda_1 D t}\sigma(0)^2. \tag{24}$$

The rate of decay only depends on the membrane diffusion coefficient D and the geometry of the membrane (through λ_1), but not on the other coefficients $C_{\mathrm{off}}, C_{\mathrm{on}}, C_{\mathrm{fb}}$.

The theorem implies that *whatever spatial features the particle density $u(x,t)$ may have will be smoothed out over time and will not return.*

Proof of the Theorem. The arguments are the same as in [**1**, Supplement]. Starting from (21) one first derives an equation satisfied by

$$v(x,t) = \frac{u(x,t) - \bar{u}(t)}{\bar{u}(t)},$$

namely,

$$\frac{\partial v}{\partial t} = \tfrac{1}{2} D\Delta v - \frac{C_{\mathrm{on}} n_c(t)}{|M|\bar{u}(t)} v.$$

Then one computes

$$\begin{aligned}\frac{d}{dt}\int_M v^2 dx &= \int_M 2vv_t dx\\ &= \int_M \Bigl\{vD\Delta v - 2\frac{C_{\rm on}n_c(t)}{|M|\bar u(t)}v^2\Bigr\}dx\\ &\le D\int_M v\Delta v dx\\ &= -D\int_M |\nabla v|^2 dx\end{aligned}$$

Since $\int_M v dx = 0$ one can apply Poincaré's inequality,

$$\int_M v^2 dx \le \frac{1}{\lambda_1}\int_M |\nabla v|^2 dx,$$

with result

$$\frac{d}{dt}\int_M v^2 dx \le -D\int_M |\nabla v|^2 dx \le -\lambda_1 D\int_M v^2 dx.$$

In view of $\sigma(t)^2 = |M|^{-1}\int v^2 dx$, this implies that

$$\frac{d\sigma^2}{dt} \le -\lambda_1 D\sigma^2,$$

and hence $e^{\lambda_1 Dt}\sigma(t)^2$ is nonincreasing. QED.

7. Derivation of (17) and competing federations of clans

Assuming that our system is between on-events we imagine the membrane particle population to be divided into clans, and we estimate the time it takes for all clans but one to have died out. As described in §5 we first group the clans together into two "federations" of clans, and denote the sizes of these federations by $n_j(t)$, $j = 1, 2$. We compute the expectation of the time at which one of the two federations has died out, i.e. at which none of the clans in one of the federations survives. At that time all membrane bound particles belong to the other federation, and thus the number of surviving clans is (at most) half the original number of clans. Repetition of the argument leads to the expected time at which the number of clans has decreased by a factor 2^k.

The federation sizes $n_j(t)$ evolve by

$$\frac{dn_j}{dt} = C_{\rm fb}(N - n_1 - n_2)n_j - C_{\rm off}n_j, \qquad (j = 1, 2) \tag{25}$$

at the ODE level. More precisely, the $n_j(t)$ evolve by a stochastic process whose generator is

$$\mathcal{L} = C_{\rm fb}(N - n_1 - n_2)\bigl\{n_1\mathcal{D}_1^+ + n_2\mathcal{D}_2^+\bigr\} + C_{\rm off}\bigl\{n_1\mathcal{D}_1^- + n_2\mathcal{D}_2^-\bigr\}. \tag{26}$$

Here the operators $\mathcal{D}_j^\pm$ are defined by

$$\begin{aligned}\bigl[\mathcal{D}_1^\pm f\bigr](n_1, n_2) &= f(n_1 \pm 1, n_2) - f(n_1, n_2),\\ \bigl[\mathcal{D}_2^\pm f\bigr](n_1, n_2) &= f(n_1, n_2 \pm 1) - f(n_1, n_2).\end{aligned}$$

To analyze the (n_1, n_2) process when N is large it is convenient to renormalize and introduce

$$x_1 = \frac{n_1}{N}, \qquad x_1 = \frac{n_2}{N}.$$

In terms of the x_j the operators $\mathcal{D}_j^{\pm}$ can be expanded in a Taylor series,

$$\mathcal{D}_j^{\pm} f = \pm\frac{1}{1!N}\frac{\partial f}{\partial x_j} + \frac{1}{2!N^2}\frac{\partial^2 f}{\partial x_j^2} + O(N^{-3}). \tag{27}$$

Using these expansions and discarding the higher order terms we can approximate the generator $\mathcal{L}$ by

$$\begin{aligned}\mathcal{L} =& \big(NC_{\text{fb}}(1-x_1-x_2) - C_{\text{off}}\big)\big\{x_1\partial_1 + x_2\partial_2\big\} \\ &+ \frac{1}{2N}\big(NC_{\text{fb}}(1-x_1-x_2) + C_{\text{off}}\big)\big\{x_1\partial_1^2 + x_2\partial_2^2\big\}\end{aligned} \tag{28}$$

The first order terms indicate that the (renormalized) clan sizes x_i evolve by a stochastic version of the ODE system

$$\dot{x}_i = \big(NC_{\text{fb}}(1-x_1-x_2) - C_{\text{off}}\big)x_i \qquad (i = 1, 2), \tag{29}$$

while the second order terms in (28) describe the stochastic fluctuations.

Under the system (29) the sum of the total membrane population, $x_1 + x_2$ converges rapidly to the equilibrium value

$$x_1 + x_2 \to x^* = 1 - \frac{C_{\text{off}}}{NC_{\text{fb}}} = 1 - \frac{n_c^*}{N},$$

which is consistent with our earlier observation that $n_c(t)$ converges to $C_{\text{off}}/C_{\text{fb}}$.

Once the total membrane population $x_1(t) + x_2(t)$ of the system has reached the equilibrium value, the deterministic model predicts that the x_i will no longer change. However, as we shall discuss in more detail below, in the stochastic model the relative magnitude of x_1 and x_2 will change due to diffusion. To analyze this drift we introduce new coordinates r, s related to x_i by

$$r = x_1 + x_2, \qquad s = \frac{x_1}{x_1 + x_2}.$$

The chain rule implies that

$$\partial_1 = \partial_r + \frac{1-s}{r}\partial_s, \qquad \partial_2 = \partial_r - \frac{s}{r}\partial_s,$$

and hence

$$\begin{aligned}x_1\partial_1 + x_2\partial_2 &= r\frac{\partial}{\partial r} \\ x_1\partial_1^2 + x_2\partial_2^2 &= r\left(\frac{\partial}{\partial r}\right)^2 + \frac{s(1-s)}{r}\left(\frac{\partial}{\partial s}\right)^2.\end{aligned}$$

In the r, s coordinates the generator $\mathcal{L}$ is therefore

$$\begin{aligned}\mathcal{L} =& \big(NC_{\text{fb}}(1-r) - C_{\text{off}}\big)r\frac{\partial}{\partial r} \\ &+ \frac{1}{2N}\big(NC_{\text{fb}}(1-r) + C_{\text{off}}\big)\left\{r\left(\frac{\partial}{\partial r}\right)^2 + \frac{s(1-s)}{r}\left(\frac{\partial}{\partial s}\right)^2\right\}.\end{aligned} \tag{30}$$

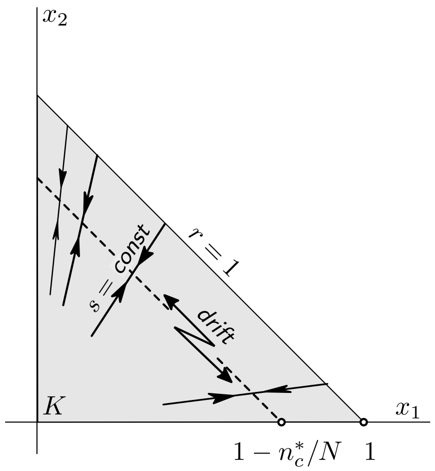

Figure 3. The (x_1, x_2) phase plane. On a short time scale (x_1, x_2) converges to the line $x_1 + x_2 = 1 - n_c^*/N$; thereafter the point undergoes a slower neutral drift along this line until it reaches either the x_1 or the x_2 axis.

We can regroup these terms as follows

$$\mathcal{L} = \frac{1}{N} A(r) \left(\frac{\partial}{\partial r} \right)^2 + B(r) \frac{\partial}{\partial r} + C(r, s) \left(\frac{\partial}{\partial s} \right)^2$$

where

$$A(r) = \frac{r}{2} \big\{ N C_{\text{fb}}(1 - r) + C_{\text{off}} \big\}$$
$$B(r) = \big\{ N C_{\text{fb}}(1 - r) - C_{\text{off}} \big\} r$$
$$C(r, s) = \frac{N C_{\text{fb}}(1 - r) + C_{\text{off}}}{2Nr} s(1 - s)$$

Since the coefficients $A(r)$ and $B(r)$ are of comparable magnitude, the expression (30) for the generator $\mathcal{L}$ of the (r, s) process shows that the dominant term is the first order term $(N C_{\text{fb}}(1 - r) - C_{\text{off}} r) r \partial_r$, while the other terms are smaller by a factor $1/N$. Thus on a short time scale (r, s) will evolve principally by the first order part

$$\mathcal{L} \approx \big(N C_{\text{fb}}(1 - r) - C_{\text{off}} \big) r \frac{\partial}{\partial r}.$$

This differential operator is a vector field. Thus on a short time scale the quantities (r, s) evolve by the deterministic system of ordinary differential equations

$$\frac{dr}{dt} = (N C_{\text{fb}}(1 - r) - C_{\text{off}} r) r, \qquad \frac{ds}{dt} = 0.$$

Figure 3 shows the flow of this system. Under this system, the fraction s of the membrane population belonging to clan 1 remains constant, while r converges to the equilibrium value

$$r = 1 - \frac{n_c^*}{N} + o(N^{-1}).$$

Once this equilibrium has been achieved, the other terms in the generator become relevant, and, on a longer time scale of order $t \sim N$, the ratio s will undergo a drift until it either hits 0 or 1. The time it takes for this to happen is the exit time. If we write $T_{\rm exit}(x)$ for the expectation of the exit time (or "mean exit time"), given that our process started at $x = (x_1, x_2)$, then $T_{\rm exit}(x)$ is the solution of an elliptic boundary value problem [**4, 5, 6**]

$$\mathcal{L}[T_{\rm exit}] + 1 = 0, \text{ on the region } K = \{(x_1, x_2) : x_i > 0, x_1 + x_2 < 1\}, \tag{31}$$

with boundary condition $T_{\rm exit} = 0$ on the exit set

$$K_{\rm exit} = \{(x_1, x_2) \in \partial K : x_1 = 0 \text{ or } x_2 = 0\}.$$

When the process starts from arbitrary (r, s) values the r component always quickly converges to the equilibrium value $1 - n_c^*/N$. One can think of the exit time as the sum of the time it takes for r to converge to the equilibrium value $1 - n_c^*/N$ and the time it takes the drift along the line $r = 1 - n_c^*/N$ to reach either $s = 0$ or $s = 1$. the first part of this evolution is only of $O(1)$, and hence negligible with respect to the total exit time. Thus we may assume, in first approximation, that the exit time only depends on s, i.e.

$$T_{\rm exit}(x) = \phi(s)$$

and that $r = 1 - n_c^*/N$. This leads to an ODE for $\phi(s)$,

$$\frac{C_{\rm off}}{N - n_c^*} s(1-s)\phi''(s) + 1 = 0, \qquad \phi(0) = \phi(1) = 0$$

whose unique solution is given by

$$\phi(s) = \frac{N - n_c^*}{C_{\rm off}} \big\{-s \ln s - (1-s)\ln(1-s)\big\}.$$

We believe this to be an accurate approximation of the true solution to (31); in any case one can show that $2\phi(s)$ is a super solution for (31), so that the true solution to (31) is bounded from above by $2\phi(s)$ (which is the direction we care about).

If one initially has $s(0) = \frac{1}{2}$, i.e. if one starts with two clans of equal size, then the expected time until one of the clans has died out is

$$T_{\rm exit} = \phi(\tfrac{1}{2}) = \frac{N - n_c^*}{C_{\rm off}} \ln 2. \tag{32}$$

By omitting the n_c^* one obtains an upper bound which is independent of n_c^*, namely

$$T_{\rm exit} \leq \frac{N}{C_{\rm off}} \ln 2.$$

8. The growth of new clans

We will now relax the assumption that there are no on events. We split the membrane bound particles in two clans, one of which contains all initially present particles and their descendants ("originals"), while the other consists of the particles which arrive through on-events and their descendants ("newcomers"). Let $n_1(t)$ be the number of newcomers at time t, and let $n_2(t)$ be the number of originals. The pair $(n_1(t), n_2(t))$ evolves by a stochastic process whose generator is the same as

in (26), except that an extra term must be included to account for the on-events. Thus (n_1, n_2) evolves by the process with generator

$$\mathcal{L}^* = C_{\text{on}}(N - n_1 - n_2)\mathcal{D}_1^+ \\ + C_{\text{fb}}(N - n_1 - n_2)\{n_1\mathcal{D}_1^+ + n_2\mathcal{D}_2^+\} + C_{\text{off}}\{n_1\mathcal{D}_1^- + n_2\mathcal{D}_2^-\}. \tag{33}$$

Setting, as before, $x_i = n_i/N$ and approximating the difference operators by differential operators, we get

$$\begin{aligned}\mathcal{L}^* &= C_{\text{on}}(1 - x_1 - x_2)\ \{\partial_1 + \tfrac{1}{2N}\partial_1^2\} \\ &\quad + \big(NC_{\text{fb}}(1 - x_1 - x_2) - C_{\text{off}}\big)\{x_1\partial_1 + x_2\partial_2\} \\ &\quad + \tfrac{1}{2N}\big(NC_{\text{fb}}(1 - x_1 - x_2) + C_{\text{off}}\big)\{x_1\partial_1^2 + x_2\partial_2^2\} \\ &= C_{\text{on}}(1 - r)\{\partial_1 + \tfrac{1}{2N}\partial_1^2\} + \mathcal{L},\end{aligned} \tag{34}$$

where $\mathcal{L}$ is the operator given by equation (30).

As in §7 we change variables, $r = x_1 + x_2$, $s = x_1/(x_1 + x_2)$. We obtain

$$\mathcal{L}^* = C_{\text{on}}(1 - r)\big[\partial_r + \frac{1 - s}{r}\partial_s\big] + \frac{C_{\text{on}}(1 - r)}{2N}\big[\partial_r + \tfrac{1-s}{r}\partial_s\big]^2 + \mathcal{L}. \tag{35}$$

As before we assume no variation in r ($\partial_r = 0$) and obtain

$$\begin{aligned}\mathcal{L}^* \approx &\frac{C_{\text{on}}(1 - r)(1 - s)}{r}\frac{\partial}{\partial s} + \frac{C_{\text{on}}(1 - r)(1 - s)^2}{2Nr}\left(\frac{\partial}{\partial s}\right)^2 \\ &+ \frac{NC_{\text{fb}}(1 - r) + C_{\text{off}}}{2Nr}s(1 - s)\left(\frac{\partial}{\partial s}\right)^2.\end{aligned}$$

As r converges to the equilibrium value

$$r = 1 - \frac{n_c^*}{N} + o(N^{-1}),$$

we substitute in

$$r = 1 - \frac{n_c^*}{N} = 1 - \frac{C_{\text{off}}}{C_{\text{fb}}N} \text{ and } 1 - r = \frac{n_c^*}{N} = \frac{C_{\text{off}}}{C_{\text{fb}}N}$$

to get

$$\mathcal{L}^* \approx \frac{C_{\text{on}}C_{\text{off}}}{C_{\text{fb}}(N - n_c^*)}(1 - s)\frac{\partial}{\partial s} + \frac{C_{\text{on}}C_{\text{off}}}{(2C_{\text{fb}}(N - n_c^*))^2}s(1 - s)\left(\frac{\partial}{\partial s}\right)^2 + \frac{C_{\text{off}}}{N - n_c^*}s(1 - s)\left(\frac{\partial}{\partial s}\right)^2.$$

The above expression can be rewritten as

$$\mathcal{L}^* \approx \frac{C_{\text{off}}}{N - n_c^*}\{s(1 - s)\partial_s^2 + \varepsilon(1 - s)\partial_s\}, \tag{36}$$

where

$$\varepsilon = \frac{C_{\text{on}}}{C_{\text{fb}}}$$

and we neglect terms of order $\mathcal{O}(N - n_c^*)^{-2}$.

In the situation we are concerned with we start the evolution with $s = 0$ at $t = 0$ (initially there are no newcomers), and we want to know how long it takes the ratio $s(t)$ to reach a certain small fraction α (we will take $\alpha = 0.1$), i.e. how much time we have before α (10%) of the membrane population can trace its ancestry to an on-event. This time is the exit time from the interval $[0, \alpha)$ through α, for the

process which starts at $s = 0$ at $t = 0$ and evolves with generator $\mathcal{L}^*$ above. The exit time is given by $\phi(0)$ where ϕ is the solution of the boundary value problem

$$-\mathcal{L}\phi = 1, \quad \phi(\alpha) = 0, \quad \phi \text{ “regular” at } s = 0, \tag{37}$$

on the interval $0 < s < \alpha$. Regularity here means that $\phi'(0)$ must exist (this is found by careful study of the generator and its possible domains). We must therefore solve the differential equation

$$s(1-s)\phi''(s) + \varepsilon(1-s)\phi'(s) = -\frac{N - n_c^*}{C_{\text{off}}}.$$

Multiplying with $s^{\varepsilon-1}/(1-s)$ and integrating twice we get

$$s^\varepsilon \phi'(s) = -\frac{N - n_c^*}{C_{\text{off}}} \int_0^s \frac{\sigma^\varepsilon}{1-\sigma} d\sigma + C.$$

The requirement that $\phi'(s)$ be finite at $s = 0$ forces $C = 0$.

Set $\sigma = s\theta$ in the integral and divide by s^ε:

$$\phi'(s) = -\frac{N - n_c^*}{C_{\text{off}}} \int_0^1 \frac{\theta^\varepsilon d\theta}{1 - s\theta}.$$

Integrating again, and using the boundary condition $\phi(\alpha) = 0$ we get

$$\phi(s) = \frac{N - n_c^*}{C_{\text{off}}} \int_s^\alpha \int_0^1 \frac{\theta^\varepsilon d\theta}{1 - \varsigma\theta} d\varsigma.$$

This integral cannot be computed in terms of elementary functions, but since α is a small number ($\alpha = 10\%$ or less), we can say that $1 - \varsigma\theta \approx 1$ with an error of at most 10%. Thus we find that

$$\phi(s) \approx \frac{N - n_c^*}{C_{\text{off}}} \int_s^\alpha \int_0^1 \theta^\varepsilon d\theta d\varsigma = \frac{N - n_c^*}{\varepsilon C_{\text{off}}}(\alpha - s).$$

Setting $s = 0$ and recalling the definition of ε we find that the expectation of the time it takes until newcomers and their descendants make up a fraction α of the total membrane population is

$$\alpha(N - n_c^*) \frac{C_{\text{fb}}}{C_{\text{on}} C_{\text{off}}} = \alpha(N - n_c^*) T_{\text{on}}, \tag{38}$$

where T_{on} was defined in (15). This is the expression we used in (16).

9. Implementation of the Model in Smoldyn

Simulations on Figure 5 (Main Text) were performed using stochastic particle simulator Smoldyn v.2.15 (http://www.smoldyn.org/).

We consider a cell of volume V in which particles transition between the active and inactive states according to the following

- Any inactive particle can spontaneously become active with rate C_{on}.
- Any active particle can become inactive with rate C_{off}.
- Any active particle can recruit any inactive particle with rate C_{fb}.

At the same time the active particles undergo Brownian motion with Diffusion rate D_A, while inactive particles diffuse with rate D_I.

We note that the model implemented in Smoldyn differs from the theoretical treatment of the positive feedback circuit described above in several ways:

(1) the rate of diffusion D_I for the inactive molecules is finite (We assume that $0 < D_A \ll D_I < \infty$).
(2) Given the bimolecular rate K_{fb}, the reactant diffusion coefficients, and the simulation time step, Smoldyn automatically computes a feedback volume V_{fb} so that at steady state the stochastic simulations agree with mass action theory.
(3) Within this small feedback volume a recruitment event occurs with probability one.
(4) Recruited molecules are offset spatially from their recruiters by a small distance in order to reduce the probability of product recombination at the next time step.

In the Smoldyn implementation the rates $C_{\text{on}}, C_{\text{off}}, C_{\text{fb}}$ are given by

(39) $$C_{\text{off}} = K_{\text{off}}\,[\text{Time}^{-1}], \quad C_{\text{on}} = K_{\text{on}}\,[\text{Time}^{-1}], \quad C_{\text{fb}} = K_{\text{fb}}/V\,[\text{Time}^{-1}].$$

Taking into account the dimensions of the parameters, the critical number of particles in the cytosol is given by

(40) $$n_c^* = \frac{C_{\text{off}}}{C_{\text{fb}}} = \frac{K_{\text{off}}\,V}{K_{\text{fb}}}$$

Note that in the Smoldyn implementation C_{fb} has no V_{fb} dependence, so equation 40 differs from equation 2. Consequently, the expected time between on events T_{on} is inversely proportional to V:

$$T_{\text{on}} = \left(n_c^* C_{\text{on}}\right)^{-1} = \frac{C_{\text{fb}}}{C_{\text{on}} C_{\text{off}}} = \frac{K_{\text{fb}}}{K_{\text{on}} K_{\text{off}} V}.$$

It follows that the requirement (19) that

$$\frac{C_{\text{on}}}{C_{\text{fb}}} \ll \frac{\alpha}{7\ln 2} (\approx 0.02 \text{ if } \alpha = 0.1).$$

becomes

(41) $$V \ll \frac{K_{\text{fb}}}{K_{\text{on}}} \frac{\alpha}{7\ln 2}.$$

Similarly, the condition for the particles of the surviving clan to be localized to a small region of the total volume becomes

(42) $$N \le N_{pol} \stackrel{\text{def}}{=} C_2 \frac{K_{\text{off}}}{D_A} V + \frac{K_{\text{off}}}{K_{\text{fb}}} V$$

in 2D geometry and

(43) $$N \le N_{pol} \stackrel{\text{def}}{=} C_2 \frac{K_{\text{off}} V^{2/3}}{D_A} + \frac{K_{\text{off}}}{K_{\text{fb}}} V$$

in a 3D volume.

As predicted by our theory, increasing number of particles throughout the simulation (see Figure 4a) we observed a transition from a homogeneous “off” state to a clustered state to a homogeneous “on” state (see Supplemental Movie 1). Keeping a fixed number of particles throughout the simulation (see Figure 4b) we observed recurrent cluster formation in the predicted regime (see Supplemental Movie 2).

We also explored the parameter space needed to observe polarization for this implementation of our model. Using the parameter values provided in the Appendix we computed the region in the (V, N) plane for which polarization occurs (Main Text Figure 5 and Supplemental Figure 3).

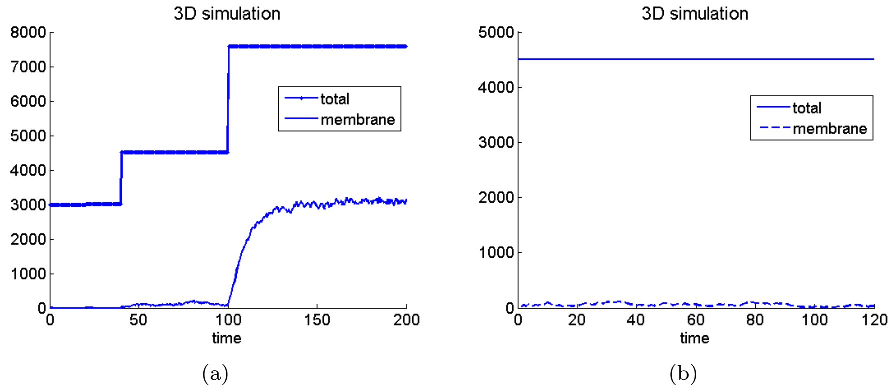

Figure 4. The total number of particles (solid curve) and the number of active particles for (a) Supplemental Movie 1, (b) Supplemental Movie 2.

Appendix A. Smoldyn Code For a Sphere ($V = \frac{4}{3}\pi L^3$)

```
# stochastic polarity model on a surface of a sphere

graphics opengl
graphic_iter 100

dim 3
species U
max_mol 20100

difc U(back) .01
difc U(bsoln) 100
color U(back) 1 0 0
color U(bsoln) 0 1 0

molecule_lists list1 list2
mol_list U(bsoln) list1
mol_list U(back) list2
display_size U(all) 5

time_start 0
time_stop 100
time_step 0.01

boundaries 0 -10 10
boundaries 1 -10 10
boundaries 2 -10 10
frame_thickness 0

max_surface 1
start_surface surf
```

```
action both U transmit
color both 0.5 0.5 0.5
polygon both edge
rate U bsoln back 0.0
rate U back bsoln 10
thickness 1
max_panels sphere 1
panel sph 0 0 0 10 30 30 s1
end_surface

max_compartment 1
start_compartment inside
surface surf
point 0 0 0
end_compartment

surface_mol 5 U(back) surf sph s1
compartment_mol 1995 U(solution) inside

reaction_surface surf kfb U(back) + U(bsoln) -> U(back) + U(back) 10
product_placement kfb unbindrad 0.16

 output_files surf2react3D_N2000_D10-2_run3.txt molpos_N2000_D10-2_run3.txt
 cmd n 100 molcountonsurf surf surf2react3D_N2000_D10-2_run3.txt
 #cmd e ifno U(back) fixmolcountonsurf U(back) 1 surf
 cmd @ 100 molpos U(back) molpos_N2000_D10-2_run3.txt

tiff_iter 1000
tiff_name surf2react3DN2000koff10kfb10D001run3_

end_file
```

Appendix B. Smoldyn Code For a 3D Domain ($V = L^3$)

```
graphics opengl
graphic_iter 100

dim 3
max_species 10
species A
species B

molecule_lists list1 list2
mol_list A list1
mol_list B list2

max_mol 6000
difc A 0.01
difc B 100
```

```
time_start 0
time_stop 100
time_step 0.0005

boundaries 0 0 10 r
boundaries 1 0 10 r
boundaries 2 0 10 r

mol 5 A u u u
mol 4995 B u u u
color A 1 0 0
color B 0 1 0

output_files phaseplane3DN5000V10_offset09.txt molpos3DN5000V10_offset09.txt
cmd n 1000 molcount phaseplane3DN5000V10_offset09.txt
cmd @ 100 listmols molpos3DN5000V10_offset09.txt

reaction off  A  -> B 10
reaction kon B -> A 0.00001
reaction bireact  A + B  -> A + A  40

#rate=40 & time_step=10^(-3) <=> binding radius=0.174157
#product_placement bireact unbindrad .175
product_placement bireact offset A 0.09 0.09 0.09

# for N>koff*V/kfb should get an endemic steady state

end_file
```

Appendix C. Smoldyn Code For a 2D domain ($V = L^2$)

```
# Simulation file for 2 interconverting species
graphics opengl
graphic_iter 50

dim 2
species U V
max_mol 20100

molecule_lists Ulist Vlist
mol_list U Ulist
mol_list V Vlist

difc U .01
difc V 10
color U 1 0 0
color V 0 1 0
display_size U 3
```

```
display_size V 3

time_start 0
time_stop 100
time_step 0.001

boundaries 0 0 40 r
boundaries 1 0 40 r

mol 10 U u u
mol 1590 V u u
cmd b pause

reaction gamma U -> V 40
reaction kon V -> U 0.00001
reaction beta U + V -> U + U 40

# for N>gamma*V/beta should get an endemic steady state
output_files outfile_SISN1600V40.txt molpos_SISN1600V40.txt
cmd n 100 molcount outfile_SISN1600V40.txt
cmd @ 100 molpos U molpos_SISN1600V40.txt
tiff_iter 5000
tiff_name 2species2DN1600kon10-5koff40kfb40V40D001_


end_file
```

References

[1] S.J., Angenent, S.B., Wang, Y., Wu, L.F., *On the spontaneous emergence of cell polarity, Altschuler,* Nature, 2008 Aug 14; 454:886-890.

[2] Bishop LM & Qian H *Stochastic Bistability and Bifurcation in a Mesoscopic Signaling System with Autocatalytic Kinase,* Biophysical Journal (2010) 98(1):1-11.

[3] *Stochastic Model for Cell Polarity,* Ankit Gupta, June 2010 http://arxiv.org/abs/1003.1404v3

[4] Geoffrey R. Grimmett and David R. Stirzaker, PROBABILITY AND RANDOM PROCESSES. Third edition. Oxford University Press, New York, 2001.

[5] N.G. van Kampen, STOCHASTIC PROCESSES IN PHYSICS AND CHEMISTRY, 1981, North-Holland.

[6] Mahnke, R., Kaupužs, J. and Lubashevsky, I. PHYSICS OF STOCHASTIC PROCESSES: HOW RANDOMNESS ACTS IN TIME, (2009) Wiley-VCH Verlag GmbH & Co. KGaA, Weinheim, Germany.
